# Exploring histone deacetylases in type 2 diabetes mellitus: pathophysiological insights and therapeutic avenues

**DOI:** 10.1186/s13148-024-01692-0

**Published:** 2024-06-11

**Authors:** Kukkala Kiran Kumar, Elhadi Husein Aburawi, Milos Ljubisavljevic, Melvin Khee Shing Leow, Xu Feng, Suraiya Anjum Ansari, Bright Starling Emerald

**Affiliations:** 1 Department of Anatomy, College of Medicine and Health Sciences, United Arab Emirates University, PO Box 15551, Al Ain, Abu Dhabi, United Arab Emirates; 2 Department of Pediatrics, College of Medicine and Health Sciences, United Arab Emirates University, Al Ain, Abu Dhabi, United Arab Emirates; 3 Department of Physiology, College of Medicine and Health Sciences, United Arab Emirates University, Al Ain, Abu Dhabi, United Arab Emirates; 4 LKC School of Medicine, Nanyang Technological University, Singapore, Singapore; 5 Dept of Endocrinology, Tan Tock Seng Hospital, Singapore, Singapore; 6 Duke-NUS Medical School, Cardiovascular and Metabolic Disorders Program, Singapore, Singapore; 7 Department of Biochemistry, YLL School of Medicine, National University of Singapore, Singapore, Singapore; 8 Department of Biochemistry, College of Medicine and Health Sciences, United Arab Emirates University, Al Ain, Abu Dhabi, United Arab Emirates; 9 Zayed Center for Health Sciences, United Arab Emirates University, Abu Dhabi, United Arab Emirates; 10 ASPIRE Precision Medicine Research Institute, Abu Dhabi, United Arab Emirates

**Keywords:** HDACs, Diabetes mellitus, HDAC inhibitors, Insulin, Pancreatic *β*-cells, Functional analysis

## Abstract

Diabetes mellitus is a chronic disease that impairs metabolism, and its prevalence has reached an epidemic proportion globally. Most people affected are with type 2 diabetes mellitus (T2DM), which is caused by a decline in the numbers or functioning of pancreatic endocrine islet cells, specifically the *β*-cells that release insulin in sufficient quantity to overcome any insulin resistance of the metabolic tissues. Genetic and epigenetic factors have been implicated as the main contributors to the T2DM. Epigenetic modifiers, histone deacetylases (HDACs), are enzymes that remove acetyl groups from histones and play an important role in a variety of molecular processes, including pancreatic cell destiny, insulin release, insulin production, insulin signalling, and glucose metabolism. HDACs also govern other regulatory processes related to diabetes, such as oxidative stress, inflammation, apoptosis, and fibrosis, revealed by network and functional analysis. This review explains the current understanding of the function of HDACs in diabetic pathophysiology, the inhibitory role of various HDAC inhibitors (HDACi), and their functional importance as biomarkers and possible therapeutic targets for T2DM. While their role in T2DM is still emerging, a better understanding of the role of HDACi may be relevant in improving insulin sensitivity, protecting *β*-cells and reducing T2DM-associated complications, among others.

## Introduction

### Diabetes mellitus: pathogenesis and complications

Diabetes mellitus (DM), caused by disruption of normal glucose metabolism, is a global problem, and its incidence worldwide is rising alarmingly, according to the International Diabetes Federation [1]. According to IDF’s projections, 643 million individuals will have DM by 2030; by 2045, the number might be 783 million.

The impairment in the function of the hormone insulin, secreted by the pancreatic *β*-cells in response to the presence of glucose is the main event in the pathophysiology of DM. The majority of people suffer from type 2 diabetes mellitus (T2DM), where changes in insulin production, secretion, and /or function change the uptake and clearance of glucose from the bloodstream and contribute to the development of T2DM. Chronic hyperglycemia that is not arrested and controlled due to impaired beta cell differentiation, proliferation and secretory dysfunction, which fail to compensate for insulin resistance, leads to microvascular complications such as end-stage renal disease (ESRD), kidney failure, and diabetic nephropathy [2], as well as inflammation of the retinal blood vessels of the eye, which leads to vision loss and diabetic retinopathy [3]. Previous research has demonstrated that ER stress, oxidative stress, and cytokine-driven inflammatory stress are all connected to the aetiology of DM at the cellular level.

### Genetic background of type2 diabetes

T2DM is polygenic and multifactorial, which involves numerous genes and their interactions, with additional contributions from environmental factors, genetics, epigenetics and lifestyle choices [4]. However, single gene mutations resulting in diabetes termed Maturity onset diabetes of the young have also been identified [5]. This involved genes such as glucokinase (GCK) [6], hepatocyte nuclear factor 1 alpha (HNF1A) [7], hepatocyte nuclear factor 1 alpha (HNF4A) [8], hepatocyte nuclear factor 1 beta (*HNF1B*) [9], sulfonylurea receptor 1 (ABCC8) [10], Insulin (INS), Neuronal differentiation 1 (NEUROD1) [11], insulin gene promoter factor 1 (IPF1 or PDX1) [12], Paired box 4 (PAX4) [13], ATP-binding cassette sub-family C member 8 (ABCC8), GATA binding protein 4 (GATA4) [14] and GATA binding protein 6 (GATA6) [15]. Potassium inwardly rectifying channel, subfamily J, member 11 (KCNJ11) [16], kruppel-like factor 11 (KLF11) [17], carboxyl ester lipase (CEL) [18], B lymphoid tyrosine kinase (BLK) [19], Adaptor protein, phosphotyrosine interacting with PH domain and leucine zipper 1 (APPL1) [20] Previous human genetic studies have demonstrated a correlation between the genetic mutations and T2DM, such as lamin A/C mutations [21], glucokinase regulatory protein (GCKR) [22], fat mass and obesity-associated gene (FTO) [23], monocarboxylate transporter 11(SLC16A11) [24], hematopoietically-expressed homeobox (HHEX) gene [25], transcription factor 7-like 2 (TCF7L2) [26], Pro12Ala polymorphism in peroxisome proliferator-activated receptor-*γ* (PPAR-*γ*) [27] and others. The role of single nucleotide polymorphisms in T2DM is also known. According to the GWAS catalogue, 5983 associations / SNPs associated with T2DM are reported (https://www.ebi.ac.uk/gwas/).

### The effect of epigenetic modifications on gene regulation and chromatin organization

In addition to the genetic makeup, epigenetic modifications also dictate phenotypic changes. Epigenetic changes are heritable transcriptional regulatory signatures without alterations in the DNA sequence but can change phenotypic plasticity by turning genes on or off transcriptionally. Epigenetic modifications, such as DNA methylation and histone post-translational modifications (PTMs), play an important role in establishing and maintaining cellular identity and also respond to changes in the environment to which the organism is exposed [28]. Histones are proteins rich in lysine and arginine residues, which play a crucial role in nuclear organization and transcriptional regulation by interacting with negatively charged DNA, forming chromatin [29]. The fundamental unit of this DNA and histone organization, termed nucleosome, consists of dimers of four histone core proteins (H2A, H2B, H3, and H4) along with an additional linker histone (H1) [30]. About 147 bp of DNA is coiled in a 1.7 superhelical twist connected to this octameric histone-DNA complex [31]. This compact arrangement of nucleosomes allows the cell to control gene expression by regulating chromatin accessibility to the transcriptional regulatory machinery [32]. The amino acids comprising core histones, especially the histone tails, undergo various reversible PTMs and play a critical role in regulating the chromatin structure and function. These PTMs, such as methylation (arginine and lysine), acetylation (lysine), ubiquitination (lysine), sumoylation (lysine), phosphorylation (serine and threonine), ADP-ribosylation and glycosylation, contribute to different regulatory mechanisms by influencing nucleosome dynamics [33]. They also regulate chromatin modifications through independent regulatory mechanisms, or crosstalk between them, termed histone code [34].

### Glucose metabolism regulation: mechanisms and disorders

Glucose is a crucial energy source for all mammalian cells and organ development, differentiation and metabolic regulation. Glycolysis plays an essential role in the breakdown of glucose for energy production. In the presence of oxygen, each glucose molecule is transformed into two pyruvate molecules, resulting in the net synthesis of two ATP molecules and two NADH molecules, which can generate further ATP via oxidative phosphorylation. In the absence of oxygen, glucose breaks down into two lactate molecules and two ATP molecules [35]. The glucose uptake, storage, and endogenous synthesis processes are tightly controlled to maintain glucose homeostasis by two major hormones, insulin and glucagon, generated by the pancreas. Insulin promotes glycolysis under high blood glucose levels, and glucagon represses glycolysis under low blood glucose levels [36]. In situations of nutrient deprivation, glucose levels are restored by the breakdown of glycogen in the liver, known as glycogenolysis [37]. The process by which glucose is produced from non-carbohydrate precursors such as amino acids, glycerol, lactate, and propionate is called gluconeogenesis, which occurs in the liver and kidneys and is essential to keep blood glucose levels steady while fasting [38]. These processes are evolutionarily conserved across various organisms, ranging from microbes to vertebrates, and their dysregulation results in hyperglycemia, characterized by elevated blood glucose levels [39]. Other hormones such as cortisol, epinephrine (adrenaline), and growth hormone are also known to affect glucose levels under stress or exercise by working antagonistically to insulin [40], disrupting glucose homeostasis resulting in chronically elevated (hyperglycemia) or insufficient (hypoglycemia) blood glucose levels when these hormones are either excessive (e.g. Cushing’s syndrome, pheochromocytoma, acromegaly) or deficient (e.g. Addison’s disease, hypopituitarism) respectively.

### From histone modifications to diabetes: the role of HDACs and HATs

**Fig. 1:**
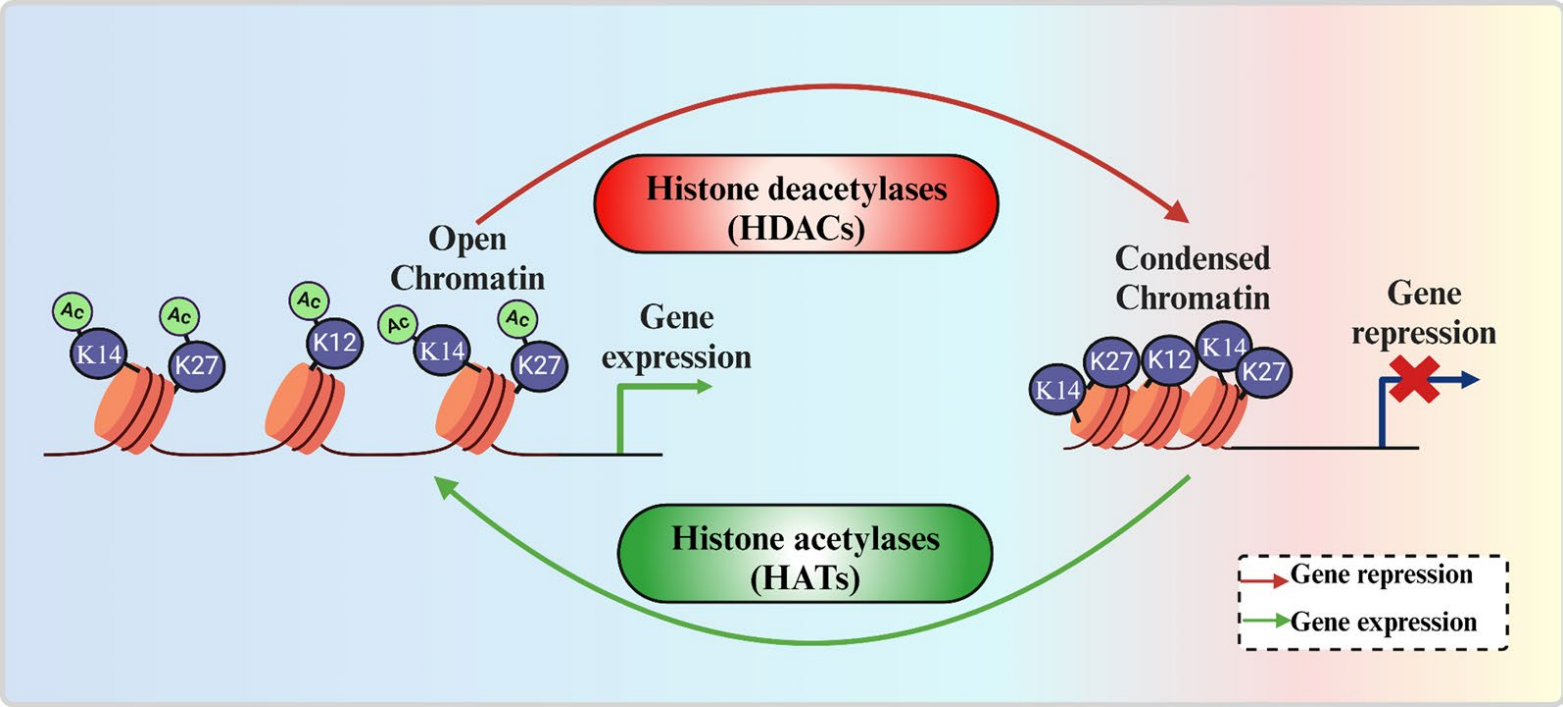
The visual representation of gene regulation through histone deacetylation and acetylation of histones by HDACs and HATs

Histone acetylation is an epigenetic PTM that affects gene expression by making genes accessible to transcriptional stimuli, resulting in gene activation [41]. Histone acetylation is controlled by two enzyme families: histone acetyltransferases (HATs) and histone deacetylases (HDACs), Fig. 1.

HATs, also called lysine acetyltransferases or KATs, use acetyl CoA as a cofactor to add an acetyl group to the ε-amino group of lysine, and neutralize the positive charge on lysine, reduce the connection between histones and DNA, resulting in transcriptional activation of genes [42]. These enzymes support numerous transcription-mediated biological activities, such as hormone signalling, cell cycle progression, and dose adjustment. HATs are classified into two types: Type A, localized to the nucleus, and Type B, localized to the cytoplasm of cells (Table 1). Type A HATs function in transcription-related histone acetylation in chromatin. Based on their homology and acetylation processes, type A HATs are classified into five groups [43]. The Gcn5-related N-acetyltransferase family includes p300/CBP-associated factor PCAF, general control of amino acid synthesis protein 5 (GCN5), and elongator complex protein 3 (ELP3) [44]. The CBP/p300 family is comprised of the CREB binding protein (CBP) and the E1A binding protein (p300) [45]. Tat-interactive protein 60 (Tip60), Monocytic leukaemia zinc finger protein (MOZ), Mortality factor on chromosome 4 (MORF), HAT binding to ORC1 (HBO1), and Tip60, monocytic leukemic zinc finger (MOZ), MOZ-related factor (MORF), human acetylase binding to ORC1 (HBO1), human males absent on the first (HMOF) are included in the MYST family [46]. TATA-box binding protein-associated factor 1 (TAF1) and Transcription initiation factor IIIC subunit 90 (TIFIIIC90) are members of the HAT family of transcriptional factors, but they are not directly involved in histone acetylation. The fifth nuclear receptor cofactors (NRCF) family has Steroid receptor coactivators (SRC) such as p600, SRC1, CLOCK, and AIB1/ACTR/SCR3 and Nuclear receptor coactivator 3 (ACTR/NCOA3) [47]. Freshly generated histones are demonstrated to be acetylated by type B HATs, which are found in the cytoplasm. Histone acetyltransferase 1 (Hat1), Hat2, HatB3.1 and Ty1 transposition 109 (Rtt109) are examples of type B HATs and are involved in DNA replication and repair processes [48, 49].

**Table 1:** The classification of HATs, their target molecules, affected molecular function and cellular location

No	HAT class	Target	Affected function	Cellular location	References
	**Type-A**				
1	Gcn5-related N-acetyltransferase family	PCAF Gnc5 ELP3	Transcription-related histone acetylation in chromatin	Nucleus	43,44,45,46,47
2	CBP/p300 family	CBP p300 PI3K Akt GLUT4 IRS1			
3	MYST family	MORF HBO1 Tip60 MOZ MORF HBO1 HMOF			
4	HAT family of transcriptional factors	TAF1 TIFIIIC90 SREBP1 Wnt			
5	Nuclear receptor cofactors (NRCF) family	p600 SRC1 CLOCK AIB1/ACTR/SCR3 ACTR/NCOA3			
	**Type-B**				
		Hat1 Hat2 HatB3.1 Rtt109	DNA replication and repair processes Acetylation of newly synthesized histones before their assembly into nucleosomes	Cytoplasm	48,49

HDACs remove acetyl groups from histone proteins [50]. HDACs and HATs are the enzymes that bring the equilibrium of histone modifications and play important roles in the remodelling of chromatin. Therefore, dysregulation of these enzymes can affect gene expression patterns and contribute to the development of different diseases, including diabetes. Recent studies have provided information on the role of HDACs in the pathogenesis and progression of DM, highlighting their potential importance as promising pharmacological targets. The link between HDACs and diabetes is multifaceted. Under conditions such as obesity and T2DM, altered HDAC activity can contribute to insulin resistance by affecting the acetylation status of transcription factors and co-regulators involved in insulin action, leading to impaired insulin sensitivity and glucose uptake [51]. Secondly, pancreatic *β*-cell function and insulin secretion are influenced by HDACs by regulating the expression of genes involved in *β*-cell development, survival, and insulin production [52]. Furthermore, HDACs are involved in other metabolic processes associated with diabetes, such as inflammation, oxidative stress, and adipocyte differentiation [53]. Therefore, the dysregulation of HDAC activity can disrupt the delicate balance of gene expression in *β*-cells, leading to impaired insulin secretion and decreased *β*-cell mass, contributing to T2DM.

### HDAC inhibitors: a promising new frontier in diabetes treatment

Regulation of HDACs using HDAC inhibitors (HDAC-i) has shown promising results in mitigating diabetes in preclinical studies and clinical trials [54]. HDAC-i are primarily epigenetic modulators, which work synergistically with other drugs to increase drug response and reduce toxicity in cancer patients, thus bringing about therapeutic advantages [55–57]. These HDAC-i have been proven to modulate gene expression patterns by affecting histone acetylation status, improve insulin sensitivity, and enhance *β*-cell function by influencing insulin receptor signalling (IRS) proteins, protein kinase B (Akt), glucose transporter 4 (GLUT4), and enzymes involved in glycolysis and gluconeogenesis [58]. Following FDA approval, two HDAC-i, vorinostat and Romidepsin, were used as antiproliferative drugs in cancer [59]. Although the use of HDAC-i is a viable option for the intervention of diabetic complications, it is crucial to note that using HDAC inhibitors in diabetic complications requires further exploration beyond their role as anticancer agents, opening up the scope of research in this domain.

This review highlights the role of HDACs in insulin resistance, expression, and secretion and discusses the potential therapeutic applications of HDAC-i in managing T2DM. Understanding the pathogenic mechanisms associated with HDAC-mediated signalling pathways may contribute to developing specific, highly potent drugs against diabetes.

## HDACs: categories

**Fig. 2:**
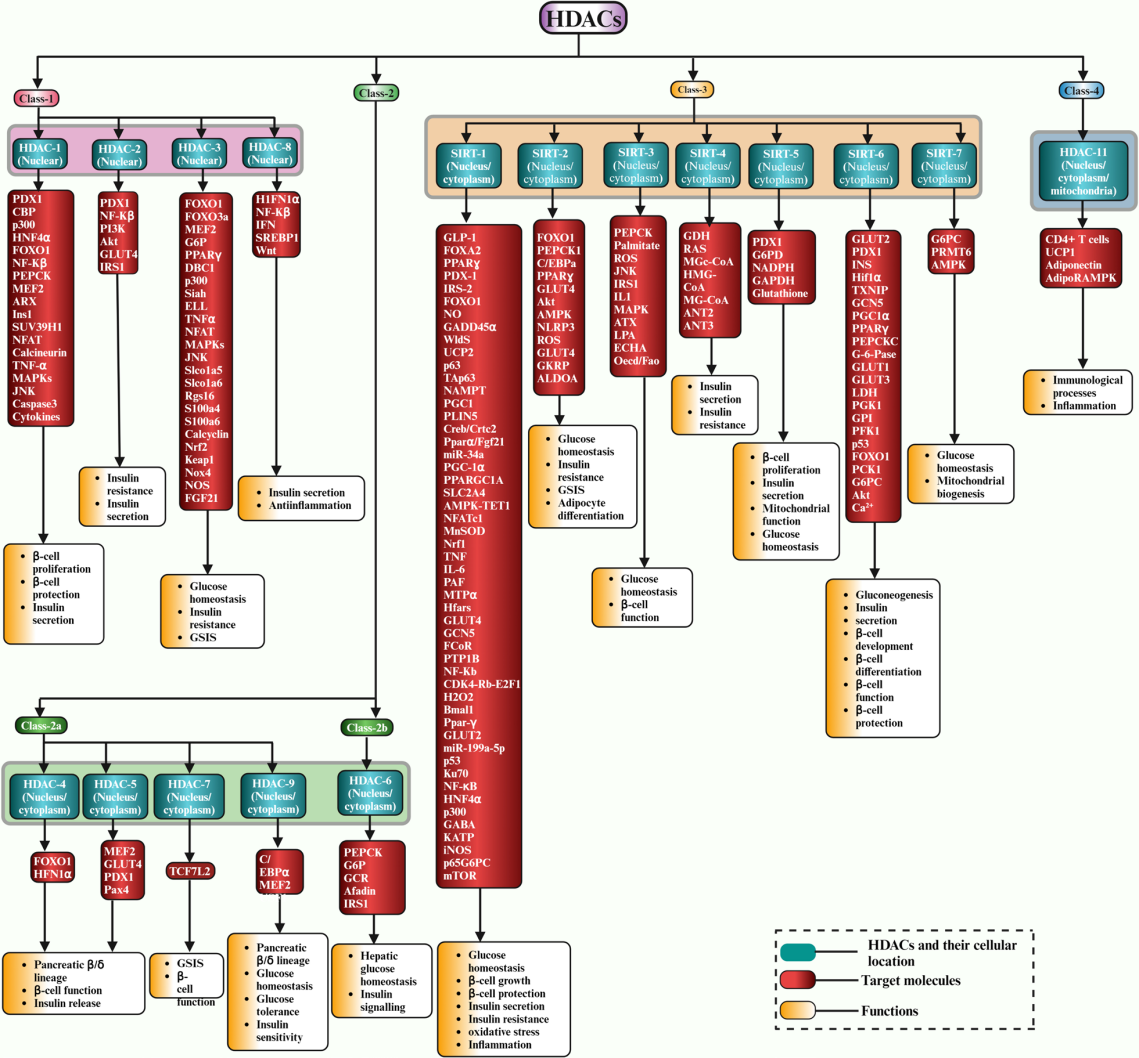
A graphical illustration of HDAC classification, cellular location, targets, and functional significance

As mentioned above, the HDAC family is a collection of enzymes that regulate gene expression by removing acetyl groups from the lysine residues of both histone and non-histone proteins and are grouped according to their sequence homology, enzymatic activity, and subcellular localization [60]. A total of 18 HDAC enzymes that deacetylate substrates containing acetylated lysines through zinc or NAD + -dependent processes were identified in humans. Class-I, II, and IV HDACs share a common catalytic mechanism incorporating a zinc ion into the catalytic processes for the hydrolysis of the acetamide bond in acetylated lysines. In contrast, Class-III HDACs require NAD^+^ as a cofactor for activity [61] Fig. 2.

Class-I HDACs, consisting of HDAC-1, HDAC-2, HDAC-3, and HDAC-8, are located mainly in the nucleus and have regulatory functions in gene regulation, cell cycle advancement, and development [62].

The Class II HDACs are divided into two subclasses: Class IIa and Class IIb. The class IIa group, which includes HDACs, HDAC-4, HDAC-5, HDAC-6, HDAC-7, and HDAC-9, can move between the nucleus and the cytoplasm and regulate gene expression in response to different stimuli such as oxidative stress, hypoxia, cellular signalling, and developmental processes [63]. They also interact with transcription factors like myocyte enhancer factor 2 (MEF2) and co-regulators such as nuclear receptor corepressor (NCoR) and silencing mediators for retinoid and thyroid hormone receptors (SMRT) to inhibit gene expression by recruiting additional chromatin-modifying enzymes or blocking the binding of transcriptional activators to control gene transcription. HDAC-6, predominantly localized to the cytoplasm, contributes to the regulation of the cytoskeleton [64].

Class-III HDACs include SIRT-uins that are different from other classes of HDACs in their structure and mechanism. They need Nicotinamide Adenine Dinucleotide (NAD +) as a cofactor for their deacetylase activity and contribute to DNA repair, metabolism, and ageing [65]. There are seven SIRT-uins, namely, SIRT-1, SIRT-2, SIRT-3, SIRT-4, SIRT-5, SIRT-6, and SIRT-7 identified in humans.

The last class IV HDACs consists of a sole member named HDAC11. This HDAC exhibits characteristics similar to Class I and Class II HDACs and also plays an essential role in managing gene expression and various immunological processes, including T-cell regulation [66] Fig. 2.

### HDACs as orchestrators of glucose metabolic balance

Maintaining steady and balanced blood glucose levels in the body is called glucose homeostasis. It involves the intricate interaction of various physiological systems that control the generation, utilization, and storage of glucose to maintain the blood glucose content within the normal range. 

#### Liver

Tight control of glucose synthesis in the liver and its uptake by peripheral tissues, such as skeletal muscle and adipose tissue, is critical to maintaining glucose homeostasis [67]. In the fed state, insulin stimulates the serine/threonine kinase protein, AKT, and activates GLUT4, causing it to move from intracellular vesicles to the plasma membrane, promoting glucose uptake [68]. The regulatory enzymes of gluconeogenesis, such as glucose-6-phosphatase (G-6-Pase) and phosphatase carboxykinase (PEPCK), are known to be suppressed by activation of AKT, which occurs by phosphorylating forkhead box protein O1 (FOXO1) [69]. During fasting, glucagon signals gluconeogenesis and glycogen breakdown in the liver [70]. It also promotes the production of PEPCK and G-6-Pase in the liver, the regulation of which requires coactivators such as Peroxisome Proliferator-activated receptor-*γ* coactivator (PGC-1a) and the C-AMP response element binding protein (CREB). Epigenetic pathways are involved in regulating glucose homeostasis, where post-translational changes significantly influence gene transcription in histones. In particular, the acetylation of histone and nonhistone proteins is crucial for regulating cellular communication and gene expression [71]. The FOXO transcription factor is crucial in limiting the expression of genes encoding G-6-Pase and PEPCK, which plays an essential role in gluconeogenesis and inhibition of class IIa HDACs (4,5,7) are shown to reduce hyperglycemia in animal models of T2DM, demonstrating their role in the regulation of G-6-Pase expression altering gluconeogenesis [72]. HDAC-1 is known to interact with hepatic nuclear factor subtype 4 (HNF4) and influences its activity by deacetylating it or its associated histones in the HNF4 response elements present in the PEPCK promoter region to stimulate liver gluconeogenesis [73]. Activation of HDAC-3 contributes to insulin resistance in T2DM by promoting FOXO1 deacetylation, which allows its interaction with DNA and gluconeogenic genes [74]. Class IIa HDACs are quickly dephosphorylated and moved from the cytoplasm to the nucleus by glucagon, where they join forces with HDAC-3 to create a complex that can deacetylate FOXO1 and FOXO3. This improves the DNA binding by the FOXO1 and FOXO3 and upregulates gluconeogenesis [75]. The orphan receptor related to the retinoic acid receptor-related orphan receptor-α (RORα) was found to play a critical role in the regulation of liver lipid homeostasis by adversely regulating the transcriptional activity of the peroxisome proliferator-activated receptor-*γ* (PPAR*γ*), which mediates liver lipid metabolism. The activation of HDAC-3 contributes to the development of insulin resistance in T2DM by promoting FOXO1 deacetylation, which allows its interaction with DNA and gluconeogenic genes [74]. Class IIa HDACs are quickly dephosphorylated and moved from the cytoplasm to the nucleus by glucagon, where they join forces with HDAC-3 to create a complex that can deacetylate FOXO1 and FOXO3. This improves the DNA binding by the FOXO1 and FOXO3 and upregulates gluconeogenesis [75]. The orphan receptor related to the retinoic acid receptor-related orphan receptor-α (RORα) was found to play a critical role in the regulation of liver lipid homeostasis by adversely regulating the transcriptional activity of the peroxisome proliferator-activated receptor-*γ* (PPAR*γ*), which mediates liver lipid metabolism. Sterol regulatory element binding protein 1 (SREBP1) was found to bind and recruit HDAC-8, which in turn activates Wingless/Integrated (Wnt) signalling components, leading to insulin resistance and hyperglycemia [76]. In obese mice, the hepatocyte NAMPT is elevated under fasting and metabolic stress, and its overexpression activates FGF21 and adipose browning, favoring glucose homeostasis and lessening dyslipidemia. Key elements of the fasting response, such as UCP1 upregulation, are regulated by hepatocyte NAMPT through its regulation of SIRT-1 [77]. The fatty acid uptake and oxidation, increased cristae packing in the mitochondria, improved systemic glucose homeostasis, and UCP1-dependent mitochondrial respiration are all mediated by increased activity of SIRT-1/PGC1 transcriptional program and lipid droplet coat protein Perilipin 5 (PLIN5) which plays a crucial role in the adaptive response of brown adipose tissue (BAT) to cold stress [78]. Systemic insulin resistance and hepatic steatosis are reduced when SIRT-1 is overexpressed in the liver of insulin-resistant mice using an adenovirus. As a result, it is hypothesized that SIRT-1 may contribute to glucose homeostasis in T2DM [79]. Eliminating the liver-specific Creb coactivator-2 (Crtc2) enhances glucose homeostasis, lowers blood glucose levels, and improves tolerance to insulin and pyruvate. In the liver, TET1 and SIRT-1 interact physically, prompting TET1 to activate the deacetylase function of SIRT-1. As a result, transcriptional factors like Peroxisome proliferator-activated receptor alpha coactivator (PGC-1α) and FOXO1 undergo deacetylation that triggers their relocation within the cell, leading to the activation of liver gluconeogenic genes such as peroxisome proliferator-activated receptor gamma coactivator 1-alpha (PPARGC1A), G6PC, and solute carrier family 2 member 4 (SLC2A4). A recent study which has looked at the function of hepatic SIRT-1 in maintaining healthy levels of lipids and glucose has shown that TET1 is a SIRT-1 coactivator and that the AMPK-TET1-SIRT-1 axis may be a therapeutic target or a mechanism for maintaining glucose homeostasis [80]. It has been shown that mice lacking liver expression of SIRT-1 exhibited mild hypoglycemia and decreased glucose synthesis. Furthermore, SIRT-1 knockdown also reduced serum cholesterol while raising levels of free fatty acids and cholesterol, suggesting that regulation of glucose metabolism in response to nutritional deprivation is significantly influenced by liver SIRT-1 [81]. The activation transcription factor 6 (ATF6) of the ER stress transducer is proteolytically broken down by glucose deprivation and enters the nucleus, where it interacts with the sterol regulatory element binding protein 2 (SREBP2) and attracts HDAC-1 that negatively regulates gene transcription and lipogenesis. Thus, HDAC-1 contributes to glucose homeostasis by being recruited by ATF6 to inhibit SREBP2-mediated gene transcription [82]. SIRT-6 encourages GCN5 activity, which acetylates PGC-1α and activates PPAR*γ* to suppress the expression of enzymes, including PEPCK-C and G-6-Pase, inhibiting liver gluconeogenesis. SIRT-6 suppresses the expression of multiple glucose-metabolic genes by deacetylating H3K9 at their promoters in mice. Under conditions of normal glucose availability, SIRT-6 represses the expression of key enzymes, diverting pyruvate to the mitochondrial TCA cycle for efficient ATP production [83]. At the promoters of glycolytic genes such as GLUT-1 and GLUT-3, lactate dehydrogenase (LDH), phosphoglycerate kinase (PGK-1), glucose-6-phosphate isomerase (GPI), and phosphofructokinase 1 (PFK-1), SIRT-6 competes with the transcriptional activator Hif1α to maintain proper glucose flux towards mitochondrial respiration and prevent excessive glycolysis [84]. In C57BL/J6 mice, the tumour suppressor p53 directly stimulates NAD^+^-dependent SIRT-6, which deacetylates FOXO1, resulting in its cytoplasmic export that down-regulates gluconeogenesis by suppression of PCK1 and G6PC [85]. Therefore, SIRT-6 is considered a master epigenetic gatekeeper of glucose metabolism. SIRT-6 overexpression has improved energy balance and glucose homeostasis in old mice. To maintain hepatic glucose production and glucose homeostasis, SIRT-6 stimulates hepatic gluconeogenic gene expression de novo NAD^+^ synthesis and systemically boosts glycerol release from adipose tissue [86]. Mutant forms of SIRT-6 with increased deacetylation activity through the enzyme evolution technique had higher deacetylation activity and improved glucose metabolism at high substrate concentrations. However, these identical mutations showed no improvement and even abrogated the SIRT-6 deacetylation activity on two specific histone PTMs, H3K9Ac and H3K56Ac, in cells. SIRT-6 has been shown to suppress the expression of glucose transporters and glycolytic enzymes in various tissues, making it a possible therapeutic target for T2DM. The E3 ubiquitin-protein ligase (Siah) uses polyubiquitylation to reduce ELL stability caused by HDAC-3-mediated deacetylation. Therefore, HDAC-3 indirectly affects glucose homeostasis by regulating the stability and function of ELL, which is involved in the expression of genes required for glucose metabolism through the DBC1 − p300 − HDAC-3 − Siah1 − ELL axis [87]. Crtc2, the liver-specific deletion reduces blood glucose levels, improves glucose and insulin tolerance, increases energy expenditure, and causes smaller lipid droplets in adipose depots. Crtc2 liver-specific knockout mice have higher plasma and hepatic.

MicroRNAs (miRNAs) are a class of small non-coding RNAs that regulate gene expression at the post-transcriptional level and play a critical role in various disease manifestations, including T2DM [88]. Fgf21 levels due to reduced miR-34a expression regulated by Creb/Crtc2 and induction of Sirt1 and Pparα. This suggests that Creb/Crtc2 negatively regulates the Sirt1/Pparα/Fgf21 axis via the induction of miR-34a under diet-induced obesity and insulin-resistant conditions [89]. Methylation of SIRT-7 at arginine 388 (R388) results in the inhibition of H3K18 deacetylase activity. Glucose availability and mitochondrial biogenesis are coordinated to maintain energy homeostasis in an AMPK-dependent way. When blood sugar levels are high, the protein arginine methyltransferase (PRMT6) blocks SIRT-7, encourages mitochondrial biogenesis, and keeps the mitochondrial respiration rate constant. Low blood sugar levels cause AMPK to hypomethylate R388, break the link between PRMT6 and SIRT-7, and maintain glucose homeostasis in an AMPK-dependent way [90]. SIRT-2 deacetylates the glucokinase regulatory protein (GKRP) and contributes to glucose regulation. It limits glucokinase's (GK) nuclear export and reduces glucose metabolism. Deacetylation of GKRP improves its stability and binding to GK [91]. SIRT-2 controls glucose-6-phosphate dehydrogenase (G6PD) activity post-translationally, which diverts glucose into the pentose phosphate pathway (PPP), where it produces nucleotides and reduced forms of NADPH and may have effects on glucose homeostasis [92].

This negative regulation of PPAR*γ* signalling through the recruitment of HDAC-3 to the PPARɣ target promoters for transcriptional repression results in dysregulation of PPAR*γ* signalling, alters glucose homeostasis and increases liver glucose and lipid metabolism [93]. The important elongation factor Eleven-nineteen lysine-rich leukaemia protein (ELL), which controls the expression of many genes, including those necessary for glucose homeostasis, maintains the stability and function of HDAC-3.

#### Adipocytes

Mice with adipocyte-specific deletion of SIRT-1 were found to be more susceptible to diet-induced insulin resistance than mice with myeloid-specific depletion of SIRT-1. SIRT-1 has been found to influence the expression and release of adipokines in adipocytes, including adiponectin, MCP-1, and interleukin 4, which modulate macrophage recruitment and polarization in adipose tissue. SIRT-1 was also shown to deacetylate NFATc1, a transcription factor that improves its binding to the IL-4 gene promoter in adipocytes, implying that SIRT-1 regulates crosstalk between adipose-resident macrophages and adipocytes, which is essential to maintain systemic glucose homeostasis and insulin sensitivity [94]. SIRT-1 can protect against metabolic side effects, such as persistent exposure to inflammation induced by a high-fat diet (HFD) and impaired glucose tolerance. SIRT-1 exerts positive effects by at least two mechanisms: up-regulation of NF-ĸB activity and elevation of the antioxidant proteins, MnSOD and Nrf1, perhaps through stimulation of PGC-1α, and reduced activation of proinflammatory cytokines, including TNF and IL-6. A clinical study has shown how platelet-activating factor (PAF) and SIRT-1 pathways interact in endothelial progenitor cells (EPCs) during disturbed glucose homeostasis in patients with T2DM. Poor glycemic management in patients with T2DM affects the amount of EPCs through altering SIRT-1 signalling via PAF receptor activation. These findings demonstrate a connection between the PAF and SIRT-1 pathways in EPCs, which adds to the detrimental impact of hyperglycemia on the functional characteristics of EPCs, which are essential in treating diabetes and peripheral vascular problems [95].

#### Muscle

The muscle glucose sensing system aids in glucose homeostasis through the Baf60c-Deptor-AKT pathway. The mechanism by which glucose causes insulin-independent AKT activation and Baf60c induction is that glucose stimulates KATP channel-dependent calcium signalling, which increases HDAC-5 phosphorylation and nuclear exclusion. Anti-diabetic sulfonylurea medications use this route to obtain their full glucose-lowering effects [96].

#### Pancreas

When blood sugar levels are high, a transcription factor is required for the formation survival of the *β*-cell mass of the endocrine pancreas and insulin gene expression, Pdx-1 interacts with HATp300 on the insulin promoter. This causes hyperacetylation (Ac) of histone H4 (H4) to increase insulin gene expression and phosphorylation of CREB, which is necessary for Pdx-1 to interact with p300. When blood sugar levels are low, Pdx-1 interacts with HDAC-1 and HDAC-2, resulting in the deacetylation of histone H4 and downregulating insulin expression. Okadaic acid treatment, which suppresses protein phosphatases, eliminates the interaction of Pdx-1 with HDAC-1 and HDAC-2 at low glucose levels, indicating the necessity of a dephosphorylation event for this interaction to occur [97]. SIRT-1 is negatively regulated by the glucoincretin hormone glucagon-like peptide-1 (GLP-1), which boosts insulin production in response to glucose and promotes the growth of pancreatic *β*-cells. In INS832/13 cells, GLP-1 elevated FOXO1 acetylation, reduced SIRT-1's affinity for FOXO1, and slowed SIRT-1 activity [98]. FOXO1 and SIRT-1 control the biological response of the pancreatic *β*-cell to nitric oxide. FOXO1 moves from the cytoplasm to the nucleus and activates transcription of the DNA repair gene, growth arrest, and the DNA damage-inducible protein 45 alpha (GADD45α), leading to FOXO1-dependent DNA repair. SIRT-1 also controls FOXO1-dependent gene expression. Nitric oxide's protective effects on cells are diminished in response to SIRT-1 inhibitors, and FOXO1 triggers a pro-apoptotic programme that results in the accumulation of PUMA mRNA and caspase-3 cleavage [99]. A fusion protein called Wallerian degeneration slow (WldS) possesses NAD biosynthesis activity that effectively prevents axon degeneration and is also associated with insulin release in *β*-cells. WldS raises pancreatic NAD levels, which interact with and increase SIRT-1 activity, and down-regulates Uncoupling protein 2 (UCP2), which controls insulin secretion [100]. The transcription factor p63 regulates glucose homeostasis by suppressing SIRT-1, and the study using hepatocyte cell lines demonstrated that the isoform TAp63 controls the SIRT-1 promoter by suppressing transcriptional activity. In obese people with T2DM, TAp63 protein levels decrease, inversely correlate with fasting glucose, and serve as a marker of insulin resistance [101]. In rats fed with HFD, the lack of SIRT-3 produces significant defects in insulin-stimulated muscle glucose uptake, resulting in an increased dependency on fatty acids. SIRT-3 may protect against dietary insulin resistance by improving glucose clearance and mitochondrial activity, thus contributing to glucose homeostasis [102]. Palmitate-induced stress of the endoplasmic reticulum (ER) in *β*-cells promotes apoptosis and diabetes, which was alleviated by overexpressing SIRT-3 cells, indicating its protective role in sustaining *β*-cell function in lipotoxic circumstances such as diabetes [103]. SIRT-3 overexpression protected high-fat diet-fed rats from glucose intolerance and insulin resistance, implying that SIRT-3 mediates processes that mitigate against an increase in enterocyte metabolic flux sufficiently to improve whole-body glucose homeostasis regardless of body weight, body composition, or fat distribution [104].

**Fig. 3:**
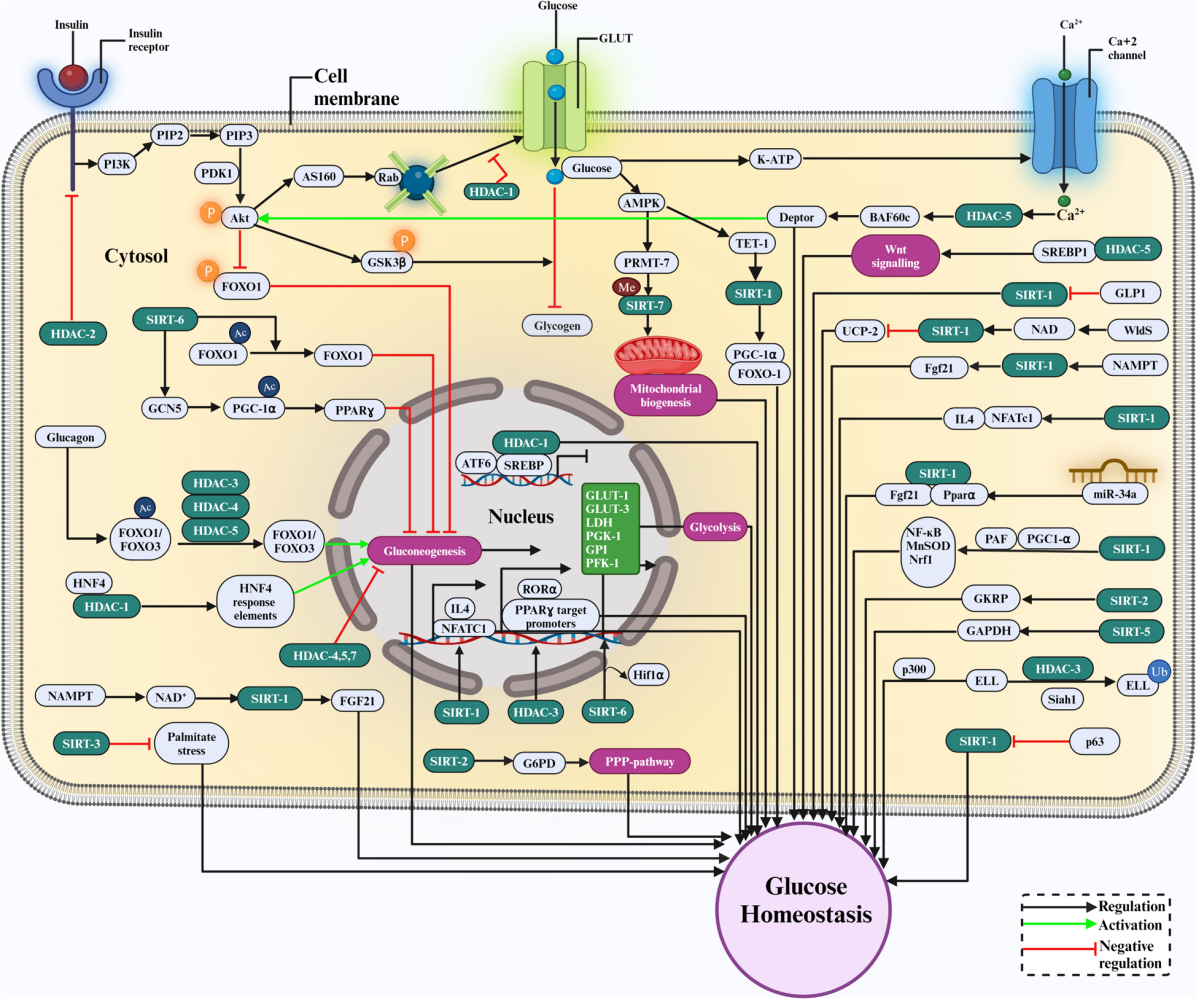
The schematic representation of the role played by HDACs for maintaining glucose homeostasis. Bright green arrows denote activation, black arrows denote regulation, and red arrows with blunt ends indicate negative regulation

The deglutarylation event activates the G6PD enzyme and increases the generation of NADPH, which is another way SIRT-5 contributes to glucose homeostasis. Maintaining glutathione in its reduced state, which aids in scavenging reactive oxygen species (ROS) and shielding the cell from oxidative damage, requires NADPH. Cells are more vulnerable to oxidative stress and cannot remove ROS when SIRT-5 is knocked down or deleted [105]. SIRT-5 was found to regulate the activity of GAPDH, a key glycolytic enzyme, through the demalonylation of a key residue, K184, located at the homodimerization interface of the enzyme that improves glycolysis [106]. The overall mechanisms of HDACs in maintaining glucose homeostasis are shown in Fig. 3.

## Decoding the link between HDACs and insulin sensitivity

Insulin resistance develops when cells become less responsive to insulin, limiting glucose absorption and utilization [107]. Several studies have mentioned the association of HDACs with the development of insulin resistance through different mechanisms, such as disruption of insulin signalling pathways, inflammatory modulation, epigenetic regulation of insulin sensitivity genes, and changes in lipid metabolism [108].

SOX6 suppresses cyclin D1 promoter activity by interacting with *β*-catenin and HDAC-1, reducing *β*-cell development by adversely regulating glucose-stimulated insulin secretion and thus contributing to insulin resistance associated with obesity [109].

HDAC-2 has been shown to bind to IRS-1 in the liver cells of db/db mice, down-regulate insulin receptor-mediated tyrosine phosphorylation of IRS-1, and decrease IRS-1 acetylation [110]. Inhibition of HDAC-2 with trichostatin-A (TSA) or HDAC-2 gene silencing enhances IRS-1 acetylation and partially attenuates insulin resistance [111]. Myocyte Enhancer Factor 2 (MEF2) regulates GLUT4 by binding to its promoter, specifically HDAC-5, limiting the transcriptional activity of MEF2 and repressing GLUT4. Also, activation of Calcium/Calmodulin-dependent protein kinase (CaMK) signalling positively regulates MEF2's transcriptional activity by causing the release of HDAC-5, which in turn activates the GLUT4 in skeletal muscle to stimulate glucose uptake, and any dysregulation leads to insulin resistance [112]. Recent studies have shown the importance of HDAC-6 in glucocorticoid receptor (GCR)-mediated effects on glucose metabolism and its potential as a therapeutic target for preventing glucocorticoid-induced diabetes [113]. For example, HDAC-6 knockout mice have been shown to have defective translocation of dexamethasone-induced liver glucocorticoid receptors. The expression of hepatic genes on dexamethasone treatment is significantly reduced in these mice. Moreover, improvements in whole-body glucose intolerance and insulin resistance were seen, indicating that HDAC-6 is a crucial regulator of insulin signalling [114]. A unique insulin-induced cellular feedback mechanism was found where the Afadin protein interacts negatively with HDAC-6 to inhibit insulin action in adipocytes [115].

Genetic ablation of HDAC-9 improves adipogenic differentiation and systemic metabolic status in HFD-fed animals, resulting in decreased weight gain, improved glucose tolerance, and insulin sensitivity. HDAC-9 knockout mice exhibit up-regulation of beige adipocyte marker genes, particularly if fed with HFD, associated with increased energy expenditure and adaptive thermogenesis, suggesting targeting HDAC-9 may be an effective strategy for combating obesity-related metabolic disease [116].

HDAC-11 activation was shown to be associated with increased cytokine levels in mouse pancreatic islets and HFD-induced hyperinsulinemia conditions, causing insulin resistance [117]. HDAC-11 is important in controlling metabolic homeostasis, and mice lacking HDAC-11 were resistant to HFD-induced obesity and metabolic syndrome, HDAC-11 depletion, improved insulin sensitivity and glucose tolerance, reduced hypercholesterolemia, and reduced hepatosteatosis and liver damage. HDAC-11 deficiency also increased energy expenditure by enhancing thermogenic ability, contributing to increased expression and activity of uncoupling protein 1 (UCP1) in brown adipose tissue. Furthermore, HDAC-11 deficiency promotes the adiponectin-AdipoR-AMPK pathway in the liver, which may contribute to hepatosteatosis reversal, suggesting it is a novel regulator of obesity with potentially significant implications for treating obesity-related diseases such as diabetes [118].

In adipocytes, activation of SIRT-1 deacetylase was shown to prevent the breakdown of the mitochondrial trifunctional enzyme component (MTPα) by lowering its acetylation and decreasing insulin resistance [119]. Mice fed phenylalanine-rich food, aspartame, or mice overexpressing human phenylalanyl-tRNA synthetase (hFARS) develop insulin resistance and T2DM symptoms. FARS phenylalanylates lysines 1057/1079 of IR (F-K1057/1079), reduces the activity of IR and inhibits insulin from stimulating cell glucose absorption. SIRT-1 reverses F-K1057/1079 and prevents hFARS and phenylalanine from inactivating insulin [120]. Endurance exercise increases insulin sensitivity in a SIRT-1-dependent way by increasing GLUT4 protein expression [121]. SIRT-1 inhibits the uncoupling of protein 2 (UCP2) in pancreatic *β* cells, promoting insulin secretion during glucose stress [122]. SIRT-1 deacetylates and activates PGC-1α, and GCN5 acetylates and represses PGC-1α, the main antagonists of mitochondrial biogenesis and function in skeletal muscle. Pharmacological stimulation of SIRT-1 in mice increases skeletal muscle expression of PGC-1α targets implicated in fatty acid oxidation, while overexpression of GCN5 lowers PGC-1α transcriptional activity [123]. Protein tyrosine phosphatase 1b (PTP1B) is known to be a significant inhibitor of the insulin receptor, and in obese, insulin-resistant mice, increased levels of PTP1B have been shown to be associated with decreased SIRT-1 expression in the skeletal muscle [124]. Eliminating SIRT-1 and GCN5 in adult mouse skeletal muscle did not improve glucose homeostasis, mitochondrial biogenesis, exercise capacity, or exercise-induced metabolic adaptation. Foxo1 corepressor (FCoR) overexpression in the white adipose tissue of Lepr db/db mice and those on a high-fat diet inhibits FOXO1 activity by increasing acetylation, which disrupts the interaction between FOXO1 and SIRT-1. As a result, this leads to decreased expression of FOXO1 target genes, reduced adipocyte size, and improved insulin sensitivity [125]. SIRT-1 also improves insulin signalling in insulin-resistant states by suppressing the expression of the protein tyrosine phosphatase 1B (PTP1B), an insulin receptor phosphatase that adversely controls insulin action [126]. SIRT-1 has a significant role in energy metabolism and NF-kB signalling linked to inflammation, which is important in obesity and insulin resistance. SIRT-2 activity is required for optimal activation of AKT by insulin and growth factors, and interaction between AKT and SIRT-2 depends on AMPK activity and involves SIRT-2 phosphorylation [127]. In macrophages, the NOD-like receptor family, the pyrin domain containing 3 (NLRP3), a pattern recognition receptor, is regulated by SIRT-2, when dysregulated, supports chronic low-grade inflammation associated with ageing and contributes to the development of insulin resistance [128].

SIRT-3 has been found to decrease mitochondrial oxidation, increase ROS, activate JNK, increase serine, decrease IRS-1 tyrosine phosphorylation, and decrease insulin signalling in cultured myoblasts [129].

Three distinct reactive species, 3-methylglutaconyl CoA (MGc-CoA), hydroxymethyl-CoA (HMG-CoA), and methylglutaryl CoA (MG-CoA), react with lysine residues during leucine oxidation to form acyl-lysine modifications which serve as the substrates for SIRT-4-mediated deacetylation. Reactive acyl-CoA species (RAS) produced during leucine metabolism interact with neighbouring proteins in the system to restrict their activity, creating a negative feedback loop that lowers pathway flux. SIRT-4 then reverses these alterations to promote leucine catabolism. The removal of SIRT-4 in mice has shown increased basal and stimulated insulin production caused by dysregulated leucine metabolism that eventually results in glucose intolerance and insulin resistance [130]. SIRT-4 has also been shown to inhibit insulin secretion, and its overexpression promotes lipogenesis and dyslipidemia, leading to insulin resistance [131]. SIRT6 overexpressing Sirt6BAC mice showed a rise in insulin-induced p-AKT/AKT ratio in the gastrocnemius muscle, improving insulin sensitivity [132]. Another study reveals histone H3 lysine 9 (H3K9) at NF-κB target gene promoters is deacetylated by SIRT6 when it binds to the NF-κB subunit RelA. This reduces the expression of NF-κB target genes, reducing inflammation in the mice's adipose tissue and HFD-induced insulin resistance [133]. Sirt6 deletion in macrophages increased NF-κB activation and interleukin-6 production, resulting in STAT3 activation and positive feedback loops for NF-κB stimulation. This led to obesity-associated tissue inflammation and insulin resistance [134].

**Fig. 4:**
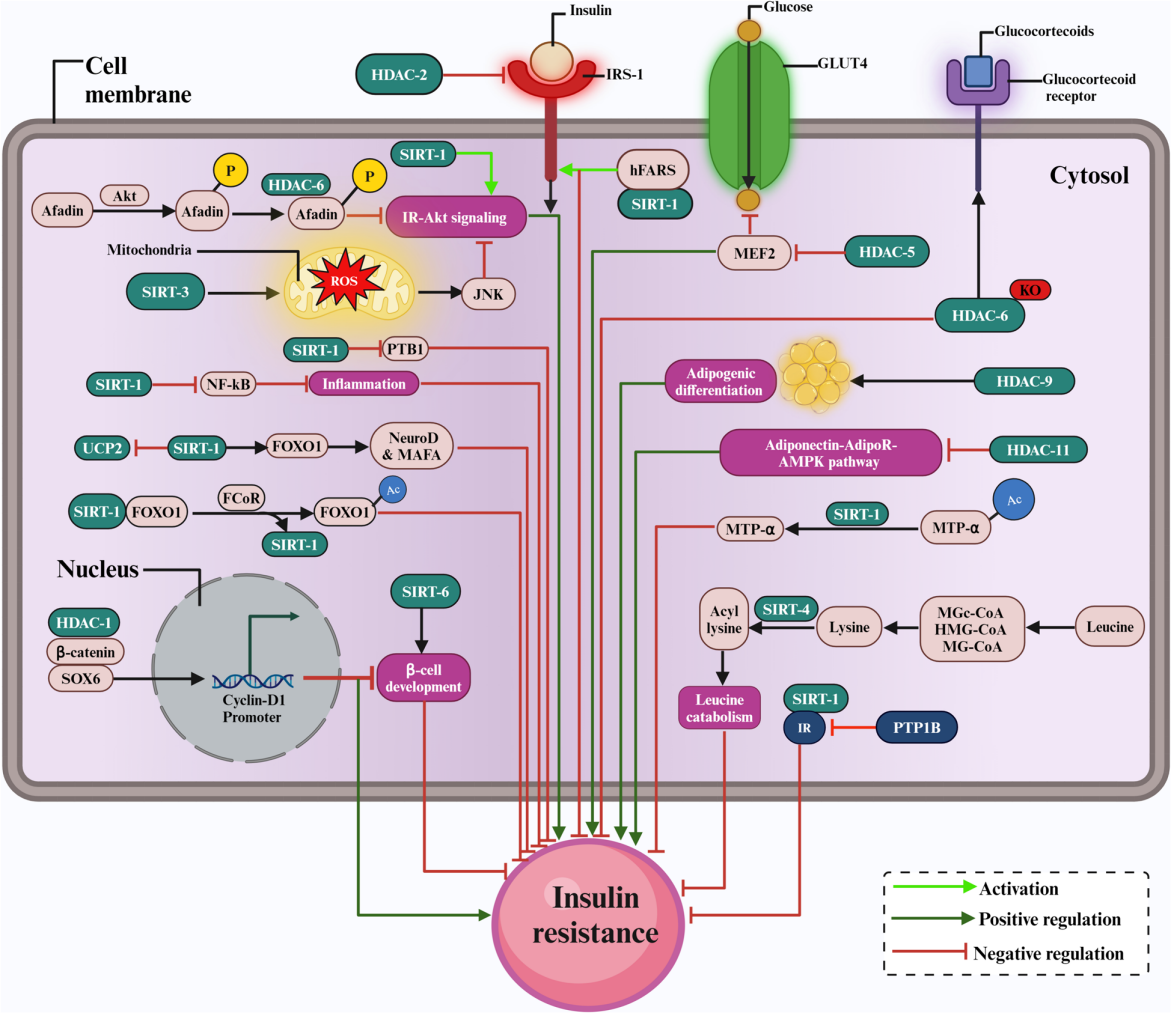
The contribution of HDACs to the development and inhibition of insulin resistance. Adipogenesis, leucine catabolism, insulin signalling, and other molecular pathways influencing insulin resistance and their interaction with HDACs. Bright green denotes activation, dark green arrows with pointed ends indicate positive regulation and red arrows with blunt ends represent negative regulation of insulin resistance

Therefore, the above analysis revealed that HDAC-1,2,5,9,11 and SIRT-3 promote insulin resistance while HDAC-6 and SIRT-1, 2,4,6 suppress insulin resistance. The contribution of HDACs in developing and inhibiting insulin resistance is shown in Fig. 4.

## Self-tolerance safeguard: HDACs influence on pancreatic *β*-cell function

Some HDACs are reported to play a protective role for pancreatic *β*-cells against cytokine toxicity by regulating Interleukin 1 (IL-1), Tumor necrosis factor (TNF) and Interferon-ɣ (INF-ɣ), which are known to promote *β*-cell death and contribute to the pathophysiology of diabetes [135]. HDAC-1, 2, and 3 have been shown to prevent cytokine-induced *β*-cell death in murine cells and increase insulin synthesis in the presence of glucose. The role of HDAC-1-PKA-Tph1 signalling in regulating *β*-cell functional compensation was also shown by activating serotonin production in *β*-cells by HDAC-1. Transgenic rats overexpressing tryptophan hydroxylase 1 (Tph1) showed increased glucose tolerance and insulin production in response to glucose. At the same time, transcriptional repression caused by HDAC-1 deacetylation of the catalytic subunit of PKA decreased its activity [136]. Mutations in HDAC-4 caused T2DM, inhibiting the deacetylation of FOXO1 and reducing *β*-cell function and insulin release. HDAC-4, HDAC-5, and HDAC-9 had restricted expression in pancreatic endocrine cells and are crucial in regulating their lineage and differentiation [137], and the overexpression of HDAC-4 and HDAC-5 decreased the number of *β*-cells [138].

HDAC-7 overexpression altered genome-wide gene expression in rat pancreatic islets and insulin-1 (INS-1) gene in *β*-cells. These changes included increased expression of the transcription factor, T-cell factor (Tcf7l2), a major transcription factor that confers diabetes susceptibility, and decreased expression of gene sets that regulate DNA replication and repair, as well as nucleotide metabolism and *β*-cell dysfunction, as seen in T2DM islets [139].

SIRT-1, 2, 3, and 6 have been shown to reduce oxidative stress and inflammation [140]. Transgenic mice overexpressing SIRT-1 showed improved glucose tolerance and increased insulin production, highlighting its importance in *β*-cell function. The nFGF1, a non-mitogenic truncation of fibroblast growth factor 1 (FGF1), can protect pancreatic *β*-cells against glucolipotoxicity-induced dysfunction and death by activating the signalling pathway of adenosine 5′-monophosphate-activated protein kinase (AMPK)/ SIRT-1/PGC-1α [141]. The circular RNA hsa_circ_0115355 prevented T2DM by enhancing pancreatic *β*-cell activity and controlling SIRT-1 expression by binding to microRNA, miR-145 [142]. The sulfated fucogalactan (SFG) protected *β*-cells from H_2_O_2_ by likely attenuating mitochondrial dysfunction via the SIRT-1-PGC1 signalling pathway, establishing SFG as a mechanistic basis for SFG as a viable therapeutic target for pancreatic *β*-cell protection [143].

IL-1 can disrupt the *β*-cell circadian clock's functionality and impede insulin production regulation. The main circadian transcription factor ARNT-Like 1 (Bmal1) and its regulator, SIRT-1, are not properly expressed, emphasizing the importance of intrinsic *β*-cell circadian clocks in regulating insulin secretion and glucose homeostasis [144]. Slc39a5, a zinc transporter, is negatively regulated in the *β*-cells of HFD-induced obese and diabetic animals. *β*-cell-specific Slc39a5 mutant animals had impaired insulin secretion, low glucose tolerance and Pgc-1α and GLUT2 expression. SIRT-1, Pgc-1, and Ppar-*γ* Agonists rescue GLUT2 downregulation in Slc39a5-deficient islets. Slc39a5-mediated zinc influx stimulates GLUT2 expression through SIRT-1-mediated Pgc-1 activation [145]. Excess circulating fat, as observed in obesity, has been shown to reduce *β*-cell function due to lower SIRT-1 activity. Following lipid infusion, rats and mice showed a significant decrease in *β*-cell function partially protected by SIRT-1 activation, which implies that SIRT-1 is a potential therapeutic target for fat-induced impairment of *β*-cell function [146]. The new polysaccharide H-1-2 from Pseudostellaria heterophylla reduced blood glucose and lipid profiles in T2DM cells, improved glucose and insulin sensitivity, and accelerated glucose-stimulated insulin secretion. Additionally, it reduced T2DM by preventing hypoxia and increasing SIRT-1 expression in isolated pancreatic *β*-cells from T2DM rats [147]. Overexpression of miR-199a-5p dramatically decreased cell viability and increased apoptosis and ROS production in INS-1 cells, accompanied by down-regulation of SIRT-1. High hyperglycemia significantly increased miR-199a-5p, while the elimination of SIRT-1 caused INS-1 cells to undergo apoptosis. SIRT-1 expression prevented miR-199a-5p from inducing ROS and attenuated high glucose-mediated apoptosis in INS-1 cells [148]. SIRT-1 was also shown to regulate the activity of transcription factors and coregulators such as p53, Ku70, FoxO1, NF-κB, PPAR*γ*, HNF4α, and p300, which are all involved in the regulation of gene expression in pancreatic *β*-cells [149].

In *β*-cell-specific SIRT-1-overexpressing (BESTO) transgenic mice, increased glucose tolerance and increased insulin production were noticed in response to glucose. During glucose and KCl stimulation, isolated BESTO islets produce more ATP, have lower UCP2, and secrete more insulin, providing a potential treatment option for T2DM [150]. In diet-induced and genetically obese mice, these molecules enhance mitochondrial capacity, decrease plasma glucose, and enhance insulin sensitivity. SIRT-1 activators improve insulin sensitivity in liver, adipose, and skeletal muscle in Zucker fa/fa rats, and glucose homeostasis offers a new viable therapeutic strategy for treating age-related illnesses, including T2DM [151].

It is well established that suppression of SIRT-3 in diabetic islets results in *β*-cell dysfunction, mediated by increased ROS generation, IL-1 synthesis, and MAPK activation. SIRT-3 mRNA expression was found to be lower in the islets of diabetic subjects compared to control groups, and suppression of SIRT-3 in INS-1 cells impaired insulin secretion and mRNA levels of several factors involved in insulin synthesis, such as MafA and PDX1, highlighting the important role in maintaining *β*-cell function [152]. SIRT-3 suppresses *β*-cell dedifferentiation by suppressing autotaxin (ATX) expression, improving lysophosphatidic acid (LPA) and regulating the ATX-LPA pathway to repair *β*-cell dysfunction caused by glucolipotoxicity [153]. Protein acetylation links fatty acid and glucose metabolism to *β*-cell activity. By lowering the acetylation level of the trifunctional enzyme subunit alpha (ECHA) in *β*-cells, the key mitochondrial deacetylase SIRT-3 prevented ECHA from degradation. When exposed to low glucose levels or during fasting, the expression of SIRT-3 was elevated in rat islets. Furthermore, when overexpressed, SIRT-3 significantly reduced palmitate-potentiated insulin production, increasing insulin secretion in islets, while SIRT-3 knockout animals had the reverse effect on fatty acid *β*-oxidation [154].

Alteration of SIRT-3 expression in *β*-cells causes widespread changes in the acetylation of important metabolic enzymes, including those involved in fatty acid oxidation [155], the tricarboxylic acid cycle (TCA) and the electron transport chain [156]. However, insulin secretion and *β*-cell metabolism are not affected without overeating. It was also found that islet function and metabolism were not significantly altered in mice with global SIRT-3 knockouts [157]. It is also shown that the reduced expression of SIRT-3 mRNA found in islets isolated from humans with T2DM, mouse islets, and INS1 cells exposed to chronic inflammatory conditions may play a significant role in *β*-cell dysfunction and T2DM through elevated levels of cellular ROS levels, IL1*β* production, and the onset of *β*-cell dysfunction.

Analysis of the function of SIRT-6 in *β*-cell development and homeostasis revealed that it suppresses the thioredoxin-interacting protein(TXNIP) expression in *β*-cells by deacetylating histone H3, which is essential for preserving *β*-cell viability and function [158]. SIRT-6 activity controls Ca^2+^ dynamics and mitochondrial function to control insulin secretion, suggesting that SIRT-6 may be a key player in controlling how mouse pancreatic *β*-cells respond to glucose by secreting insulin [159].

SIRT-6 levels were reduced after exposure to palmitate, causing the *β*-cells to become dysfunctional and eventually die. SIRT-6 is a protein that regulates insulin production in response to glucose stimulation in pancreatic *β*-cells. Even in the absence of palmitate exposure, eliminating SIRT-6 in MIN6 cells increased cell mortality and reduced insulin production. Thus, SIRT-6 is protective against palmitate-induced *β*-cell dysfunction and mortality [160].

The FOXO1 protein prevents pancreatic *β*-cells from failing due to oxidative stress by promoting the expression of NeuroD and MafA transcription factors, acts as a bridge between glucose and growth factor receptor-activated pathways, and shields *β*-cells from oxidative damage [69]. HDAC inhibitors (HDACi) affect pancreatic *β*-cells function by regulating miRNAs, and HDACs are required to establish appropriate ratios of *β*-cells and δ-cells [161].

**Fig. 5:**
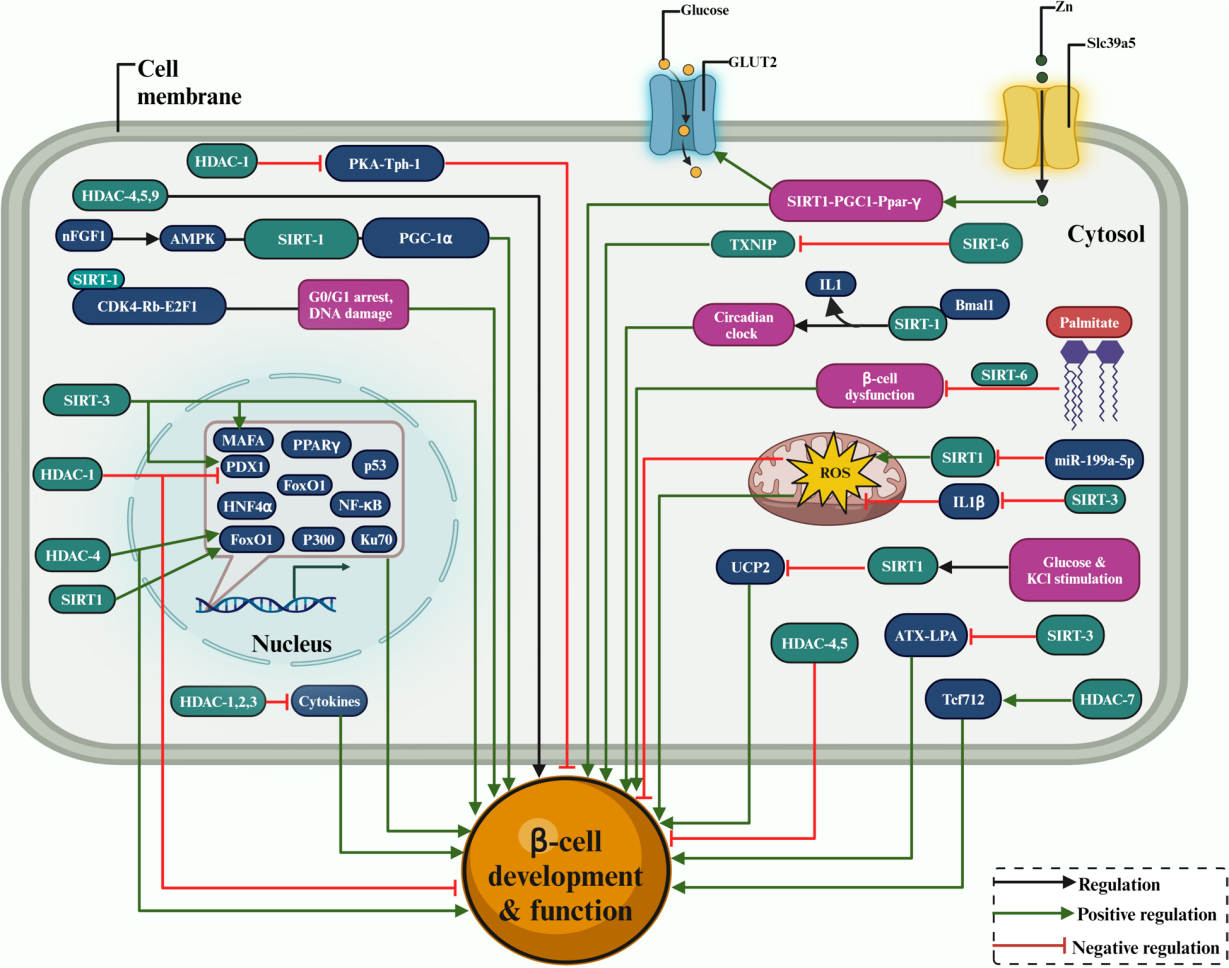
The role of HDACs in developing, protecting, and inhibiting pancreatic *β*-cells. The regulatory contribution to pathways such as cell cycle, glucose and KCL stimulation, SIRT1-PGC1-Pparɣ pathway, transcriptional regulation in coordination with GLUT transporter, and zinc transport (Slc39a5) inhibition are shown. Dark green denotes activation, black arrows denote regulation, and red arrows with blunt ends denote negative regulation

Therefore, HDAC-2,3,7 and SIRT-1,3,6 have positively contributed to *β*-cell maintenance, function, and protection, while HDAC-4,5 negatively regulates *β*-cell differentiation. HDAC-1 has both positive and negative effects on maintaining the functionality of *β*-cells. The overall functional significance of HDACs in the development and function of pancreatic *β*-cells is summarized in Fig. 5.

## Fine-tuning insulin dynamics: the intricate functionality of HDACs

Pancreatic *β*-cells release insulin in response to acetylation-regulated nutrient stimulation and maintain glucose homeostasis [162]. Insulin plays a supportive role in fed-state glycolysis, glucose absorption, and glycogen synthesis. Unbalanced glucose uptake by skeletal muscle encourages excess glucose to return to the liver, resulting in a higher load of free fatty acid circulation and fat deposition, leading to insulin resistance and T2DM [163]. HDACs can inhibit insulin production and secretion through the downregulation of transcription factors, including PDX1, the homologue of the V-maf musculoaponeurotic fibrosarcoma oncogene A (maf-A), and neurogenic differentiation 1 (NeuroD1) [164]. The acetylation of histone H4 was shown to have a regulatory role in insulin expression. PDX1 interacts with CBP and HAT called p300 to increase proinsulin expression, while HDAC-1 can inhibit this interaction, leading to *β*-cell malfunction and repression of insulin expression [69].

Menin and glucagon-like peptide-1 (GLP-1) pathways are both important in the *β*-cell function where menin is directly phosphorylated at serine 487 by GLP-1 signalling-activated protein kinase A (PKA), releasing the inhibition of insulin production and *β*-cell proliferation caused by menin. Increased Ins1 gene transcription also coincides with the decrease in the binding of the restrictive epigenetic histone modifier, suppressor variegation 3–9 homologue protein 1 (SUV39H1) and HDAC-1 at this locus [165]. In response to calcium signals resulting from metabolic and inflammatory stress, the nuclear factor of activated T cells (NFAT) interacts with calcineurin to regulate the genes in pancreatic islets. To differentially regulate the insulin and TNF-α genes, the NFAT directs MAPKs, p300, HDAC-1 and HDAC-3 to gene promoters. In response to glucagon-like peptide 1, NFAT and ERK bind to the insulin gene promoter, but in response to IL-1, NFAT forms complexes with p38 MAPK (p38) and Jun N-terminal kinase (JNK) on the promoters of the TNF-α gene. Complex stability requires MAPK activity, and translocation of NFAT and MAPKs to gene promoters relies on calcineurin/NFAT [166].

HDAC-3 controls insulin secretion, and animals lacking the HDAC-3 gene showed significantly increased glucose tolerance and higher basal and glucose-stimulated insulin production. Numerous organic anion transporter genes, such as Slco1a5 and Slco1a6, Rgs16, S100a4, S100a6 and transporter Rgs16, have contributed to insulin release. The Slco1a6 gene locus has been recognised as a significant quantitative trait locus (QTL) in *β*-cells that controls gene transcription by modifying bile acid transport in pancreatic islets. It was demonstrated that bile acids enhance insulin secretion induced by glucose, and Rgs16 increases insulin secretion induced by glucose. S100a6, also known as calcyclin, has been found to improve Ca2 + -stimulated insulin release in response to glucose through the regulation by HDAC-3 [167].

SIRT-1 inhibited oxidative stress-induced hyperglycemia and cytokine toxicity on *β*-cells by deacetylating FOXO1 and the NF-kB component p65, thereby protecting insulin secretion [69]. SIRT2 deletion in rat *β*-cells resulted in decreased glucose-stimulated insulin secretion (GSIS) and impaired glucose tolerance without compromising insulin sensitivity by promoting aldolase (ALDOA) degradation and preventing the degradation of glucokinase regulatory protein (GKRP) [168].

SIRT-4 inhibits GDH activity in the mitochondria by ADP-ribosylation, lowers insulin release in response to amino acids, and lessens these effects by calorie restriction [131]. SIRT-4 inhibits glutamate dehydrogenase activity through ADP-ribosylation and controls insulin secretion in *β*-cells. GDH converts the glutamate generated after the *β*-cell is exposed to glutamine to the TCA cycle intermediate α-ketoglutarate, depending on the cell's energy state. This encourages ATP production, insulin secretion, and mitochondrial activation. Allosteric effectors tightly regulate the activity of GDH, leucine, and ADP stimulate it while GTP inhibits it.

Additionally, SIRT-4-mediated mono-ADP-ribosylation of GDH is a posttranslational modification that limits amino acid-stimulated insulin release by inhibiting GDH function [169]. In mice, nutrient-stimulated insulin production was unaffected by SIRT-4 ablation, a protein that controls cellular stress and ageing. This shows that SIRT-4 may not be a major factor in insulin release by mouse *β*-cells [170]. Earlier studies have shown that SIRT-6 activation can enhance glucose-induced insulin secretion, while SIRT-4, 5 and 7 are believed to affect insulin secretion, contributing to T2DM [171]. SIRT-4 controls insulin secretion and increases insulin production in response to glucose, and the ADP/ATP carrier proteins ANT2 and ANT3 interact with SIRT-4 to regulate insulin secretion [172].

SIRT-6 knockout mice (S6KO) showed substantial impairments in glucose-stimulated insulin secretion (GSIS) and acquired glucose intolerance. It is well known that SIRT-6 deacetylates FoxO1, altering its nuclear export and releasing its transcriptional repression of key glucose-sensing genes such as PDX1 and GLUT2. At the same time, SIRT-6 overexpression in the S6KO islets restored PDX1 and GLUT2 expression and rescued defective insulin secretion. These studies have demonstrated how SIRT-6 in pancreatic cells deacetylates FOXO1 and then increases the expression of PDX1 and GLUT2 to preserve the ability of the pancreatic cells to sense glucose and maintain glucose tolerance [173]. GSIS was significantly reduced in MIN6 *β*-cells after SIRT-6 knockdown. The islets of SIRT-6 knockout mice had lower levels of ATP, lower mitochondrial oxygen consumption, lower cytosolic calcium dynamics in response to glucose or potassium chloride, more damaged mitochondria, and lower levels of the mitochondrial complex [159].

**Fig. 6:**
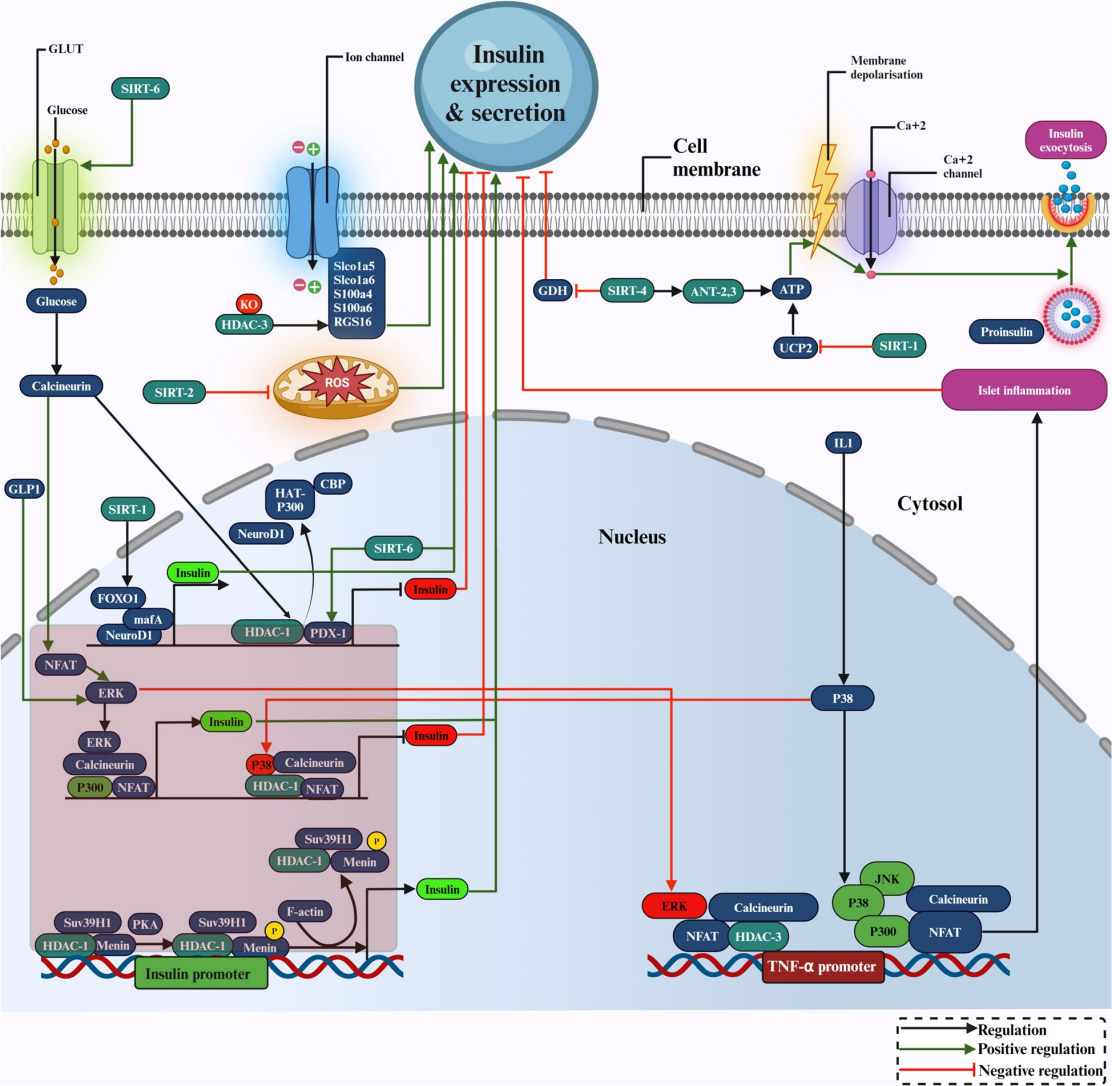
The diagrammatic portrayal of HDACs' roles in the transcriptional regulation of insulin promoters, ROS production, ionic transporters, and leucine catabolism are all associated with insulin expression and secretion. Bright green arrows denote activation, black arrows denote regulation, and red arrows with blunt ends indicate negative regulation

In general, HDAC-1,3 and SIRT-4 have shown adverse effects on insulin secretion, but SIRT-1, SIRT2, and SIRT-6 have improved insulin secretion and glucose uptake. The involvement of HDACs in the transcriptional regulation of insulin and TNFα promoters for their expression, islet inflammation, ROS generation, and ion transporters is shown in Fig. 6.

## Diabetes intervention strategies: HDAC inhibitors in focus

HDAC inhibitors (HDAC-i) are the molecules that promote histone acetylation by inhibiting the function of HDAC enzymes. Depending on the genes implicated, this acetylation can have a variety of impacts on gene expression. HDAC-i encourages an open chromatin state, which leads to enhanced expression of specific genes, such as those involved in regulating cell differentiation, apoptosis, and cell cycle control. As HDACs have also been shown to play an effective role in a variety of diseases, including cancer, diabetes, neurological, inflammatory, hypertension, angiogenesis, and vascular problems, HDAC-i serves as a selective tool for regulating HDACs and to study their effect on diseases by analyzing chromatin remodelling [174]. Several studies have shown the efficacy of HDAC-i such as trichostatin A (TSA), suberanilohydroxamic acid (SAHA), valproic acid (VPA), vorinostat, govinostat/givinostat (ITF2357), or entinostat (MS275), artesunate (ART), mocetinostat (MGCD0103), N-acetyldinaline (CI-994), resveratrol, MC1568, BRD3308, sodium butyrate (NaB), vaccarin, tubastatin-A against diabetes. The list of HDAC-i and their structures are shown in Table 2.

**Table 2:**
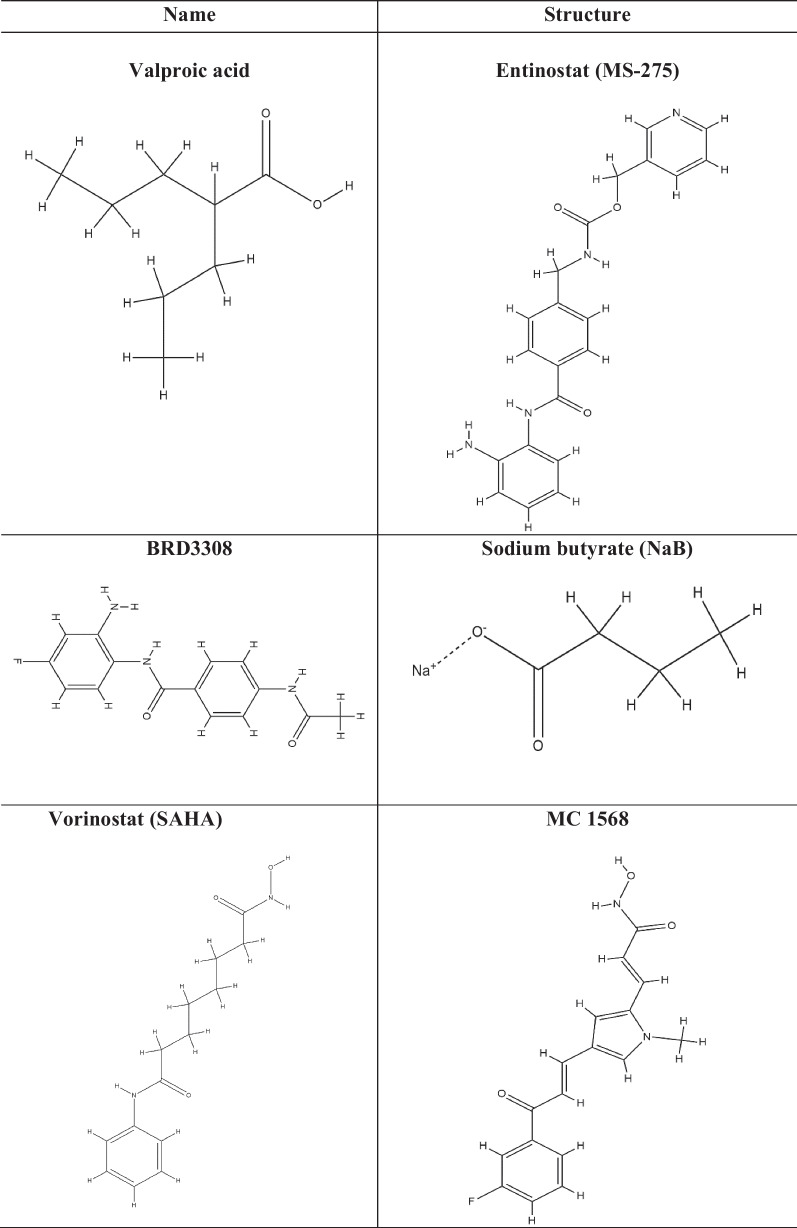

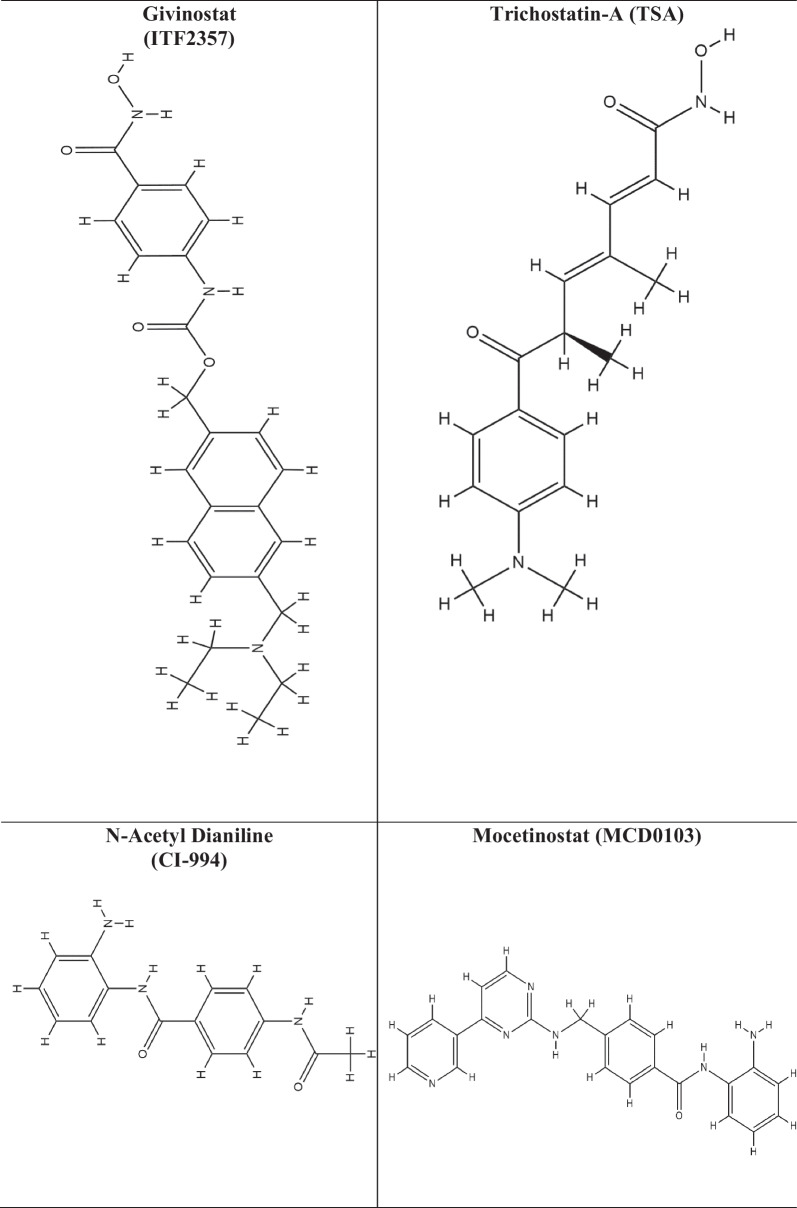

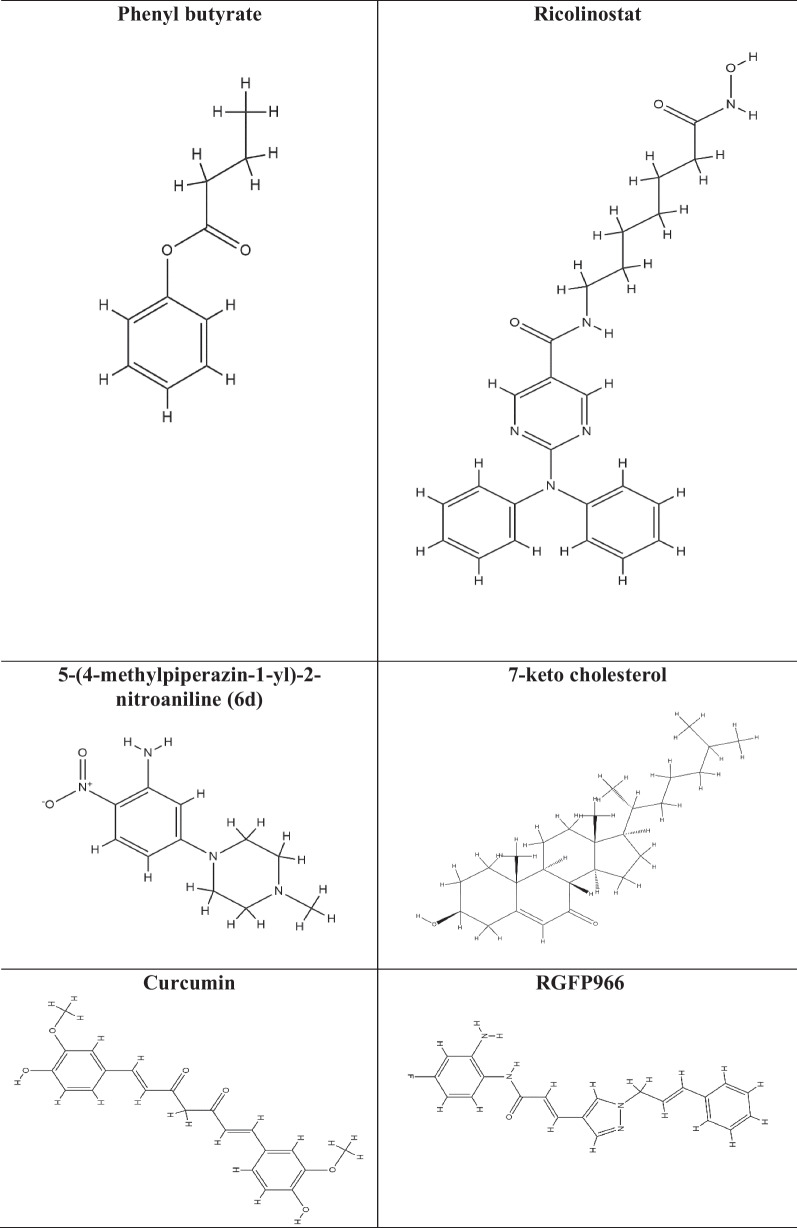

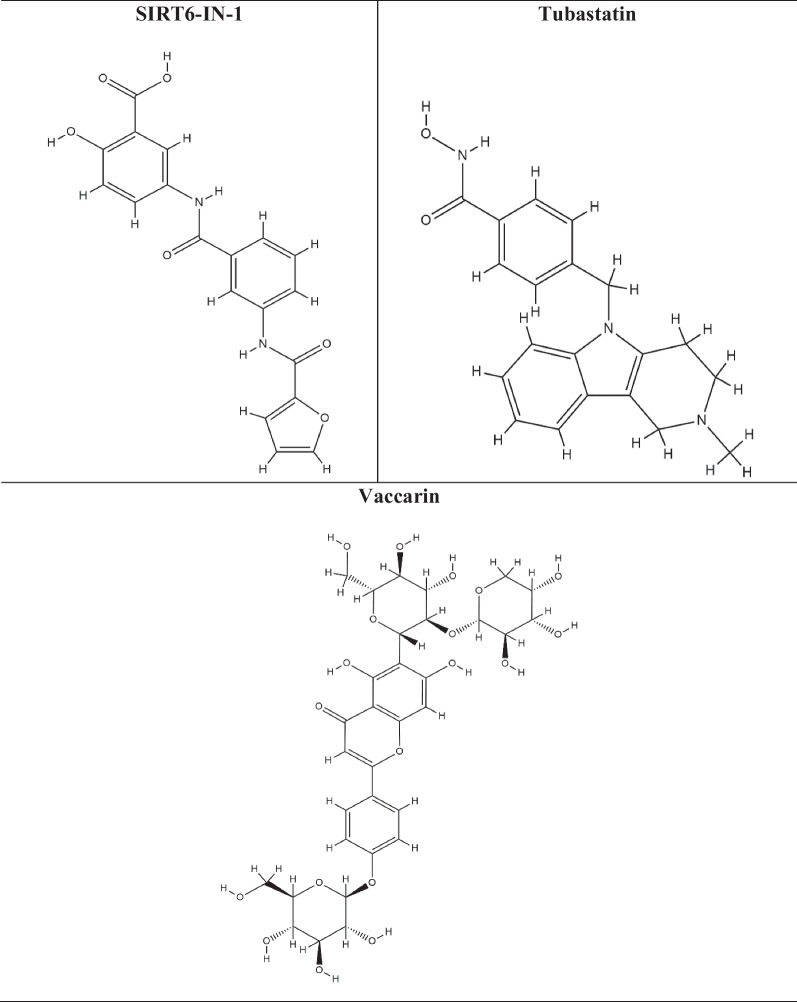
List of HDAC inhibitors (HDACi) and their structures

Vaccarin (VAC), is an active flavonoid glycoside derived from vaccariae semen. In human microvascular endothelial cells (HMEC-1), HG treatment lowered cell viability, increased HDAC-1 protein levels, boosted ROS formation, induced cell cycle arrest, and induced cell death, and all these were reversed by VAC treatment, suggesting that VAC could treat diabetes-related endothelial dysfunction [175].

The protective effect of inhibition of HDAC-3 by inhibitor RGFP96 can be attributed in part to the activation of Nrf2 signalling by suppressing Kelch-like ECH-associated protein 1 (Keap1) levels, modulation of the Nox4-Nrf2 redox imbalance, and inhibition of endothelial NOS uncoupling suggesting the role of HDAC-3 as an epigenetic regulator in T2DM related vasculopathy [176]. In T2DM db/db mice, inhibition of HDAC-3 altered the miR-200a/Keap1/Nrf2 signalling pathway and the expression of the downstream targeting junction protein, a possible new therapeutic target for degradation of the T2DM-related blood–brain barrier (BBB) [177]. RGFP966 inhibited HDAC-3 and prevented diabetic aortic pathologies in male OVE26 type 1 diabetic mice compared to wild-type (FVB) mice. Furthermore, inhibition of HDAC-3 by RGFP966 dramatically reduced aortic fibrosis and inflammation in OVE26 mice. Also, inhibiting HDAC-3 may activate Nrf2, reducing diabetes-induced liver damage and plasma fibroblast growth factor 21 (FGF21) production and secretion, offering aortic protection [178].

HDAC-i, VPA, was shown to aid the development of the pancreatic endoderm by increasing PDX1 expression, improving *β*-cell differentiation and function, and suppressing TGF signalling to cause α-cell to *β*-cell trans-differentiation [179]. It also improved the functionality and lifespan of fresh pancreatic islets used in islet transplantation to treat hyperglycemia [180]. In newly diagnosed epileptic patients, it lowered hyperinsulinemia [181]. VPA increased insulin secretion in vitro when the treated islet cells from donors were used [182].

A broad spectrum HDAC-i, SAHA, also called vorinostat, was demonstrated to influence the development of *β*-cells in a mesenchymal stem cell model, and upon treatment with SAHA, mesenchymal stem cells MG63 showed enhanced insulin production with an increased transcription factor, PDX1 expression. Also, pretreatment with SAHA showed an elevation in the markers of *β*-cell when challenged with high glucose, suggesting its potential to enhance stem cells capacity to differentiate into insulin-producing *β*-cells [183]. This HDAC-i was the first approved by the FDA to treat Cutaneous T-cell lymphoma (CTCL) [184]. In skeletal muscle, SAHA prevented lncRNA H19 inhibition and increased HDAC-6 activity associated with downregulating IRS1 levels [185].

CI-994 is another HDAC-i derived from benzamide for HDAC-1, HDAC-3, HDAC-6, and HDAC-8, known for its anti-inflammatory potential in DM and also improved pancreatic polypeptide cells or α-cell enlargement and minimised cytokine-induced apoptosis in *β*-cells [186]. The variant CI-994 is a highly potent and selective HDAC-3 inhibitor. It has been shown to reduce pancreatic *β*-cell death caused by inflammatory cytokines and gluco-lipotoxic stress while increasing functional insulin output.

Adipose tissue produces the hormone leptin, which can reduce food intake and increase energy usage. HDAC-6 inhibitors have potent leptin-sensitizing and anti-obesity effects. The HDAC-6 inhibitor Tubastatin A improves systemic glucose homeostasis. It reduces food consumption, fat mass, liver steatosis, and food intake [187].

HDAC-i, NaB, reduces hyperglycemia, lowers blood cholestenone (TC) and low-density lipoprotein (LDL-c), and improves insulin resistance and glucose tolerance. NaB also improves diabetes-induced histological changes in islets and functional impairment. NaB inhibited the expression of ERS-related proteins such as phosphorylated type I transmembrane ER-resident protein kinase (p-PERK), phosphorylated eukaryotic initiation factor 2 (p-eIF2), activating transcription factor 4 (ATF4), and CCAAT/enhancer-binding protein homologous protein (CHOP) thus protecting against T2DM by blocking the PERK-CHOP pathway of ERS [188]. Treating juvenile diabetic rats with NaB enhanced *β*-cell proliferation, function, and glucose homeostasis while decreasing *β*-cell death by modulating the p38/ERK MAPK and apoptotic pathways [189]. NaB treatment reduced plasma glucose, HbA1c, and pancreatic beta-cell mortality in diabetic mice. It also increased insulin levels, which helped to maintain glucose homeostasis. Additionally, it turned on the protein Nuclear factor erythroid 2-related factor 2 (NRF2), which guards cells against oxidative stress. NaB-treated diabetic mice without the Nrf2 gene for 20 weeks exhibited severe kidney damage, inflammation, apoptosis, fibrosis, and albuminuria, which was protected by NaB treatment and decreased HDAC activity, elevated the expression of Nrf2 and its downstream targets, heme oxygenase 1 (HO1) and NAD(P)H dehydrogenase quinone 1 (NQO1) [190].

A selective class IIa HDAC-i, MC1568, has been shown to control the development of endocrine cells. Complete knockouts of HDAC5 and HDAC9 in mice increased the number of *β*-cells that produce insulin. In contrast, knockouts of HDAC-4 and HDAC-5 increased δ-cells that produce somatostatin. On the other hand, HDAC-4 and HDAC-5 overexpression resulted in a decreased pool of *β*- and δ-cells. It suppressed the MEF2 target genes, PDX1 and activated paired box-4 (Pax4) in pancreatic progenitors and increased the number of endocrine *β*-cells and δ-cells, suggestive of a route for *β*-cell differentiation-based therapies for T2DM [138]. The expression of HDAC-7 was higher in T2DM patient’s pancreas and has been associated with a decrease in insulin production from *β*-cells, MC1568 significantly increased glucose-stimulated insulin production in T2DM donor islets without affecting non-diabetic donor islets and also increased clonal *β*-cell insulin secretion, mitochondrial respiration, and ATP content while lowering *β*-cell apoptosis [191]. This suggests HDAC-7 inhibition by MC1568 could be a potential therapy for T2DM.

ITF2357 is known to target class I and II HDACs. It outperformed SAHA in anti-inflammatory efficacy in both in vivo and in vitro studies and has been shown to increase *β*-cell survival at clinically relevant doses during inflammatory circumstances and has therapeutic potential in treating T2DM [192]. It saved *β*-cells from cytokine-induced cell death and restored insulin secretion by regulating HDAC-1, HDAC-11, HDAC-2, and HDAC-6 [117]. Aberrant bone marrow-derived cells (BMDCs) enhance diabetic complications, and removing these cells in diabetic mice with bone marrow transplantation resulted in better control of their glucose levels. In the diabetic mouse model, aberrant BMDCs with epigenetic changes are treated with the ITF2357. Even after stopping insulin and givinostat, those mice are normoglycemic and have regained insulin secretion. In clinical medicine, combo therapy has been proposed for patients striving for complete diabetic remission [193].

Mocetinostat, an orally active HDAC-i benzamide isotype, is reportedly potent against HDAC-1 together with HDAC-2, HDAC-3 and HDAC-11 [194]. It has improved diabetes by increasing specificity protein, Krüppel-like factor (SP1) acetylation and enrichment of superoxide dismutase promoters. It has also been shown that treatment with MGCD0103 effectively protects pancreatic *β*-cells [195].

HDAC-i, MS275 inhibits HDAC-1, 2, and 3 and reduces the activity of the pro-apoptotic protein caspase-3 [196]. It selectively inhibited HDAC-3 and prevented *β*-cell death caused by cytokines. Treatment with the selective HDAC-3 inhibitor MS-275 treatment and suppression of HDAC-3 was able to stop cytokine-induced *β*-cell death [197]. Targeting HDAC-3 with MS-275 protects the pancreatic *β*-cells from lipotoxicity by decreasing the palmitate-induced apoptosis. It attenuates the expression of endoplasmic reticulum stress markers like activating transcription factor 3 (Atf3) and CAAT/enhancer-binding protein homologous protein-10 (Chop), which are induced by palmitate and contributes to *β*-cell death [198].

Curcumin reduces hydrogen peroxide (H_2_O_2_)-induced cell death in pancreatic *β*-cells, where it blocks the SIRT-1-mediated protein kinase and the stress pathway ER kinase (PERK)-C/EBP homologous protein (PERK-CHOP) [199].

Resveratrol is a naturally occurring polyphenol-derived substance in red grapes [200]. It is a class III HDAC activator and has been reported to activate SIRT-1 and SIRT-2. SIRT-1 activation has been shown to protect against pancreatic *β*-cell damage by counteracting NF-ĸB [135]. Stimulation of SIRT-1 by gamma-aminobutyric acid (GABA) has promoted cell proliferation [201]. It may enhance insulin sensitivity and control the generation of glucose by activating SIRT-1 in the hypothalamus and modifying the activity of KATP channels and hepatic vagal tone [202]. Compared to control islets, nitrite production and iNOS expression were lowered by overexpressing SIRT-1 and pretreatment of the islets with resveratrol by inhibiting iNOS expression and NF-κB signalling by deacetylation of p65 [135]. Enhancement of SIRT-1 activity by resveratrol or SIRT-1 overexpression enhanced *β*-cells tolerance to cytokine toxicity and increased the viability of islet cells. It also promoted longevity and improved glucose homeostasis in mice by stimulating the SIRT-1-mediated deacetylation of the transcriptional coactivator PGC-1α [203]. In humans, the shape of the islets incubated with HFD + resveratrol was identical to that of the control islets. With an HFD and an HFD + resveratrol diet, insulin secretion increased 2.6 and 6.2 times, respectively, while EX-527, a specific SIRT-1 inhibitor, markedly reduced insulin secretion, suggesting that SIRT-1 upregulated insulin secretion. Furthermore, resveratrol administration increased the expression of *β*-cell-specific transcription factors, PDX1, NKX6-1, FOXO1, and SIRT-1, in human islets [204].

The protective effects of HDAC-i, ART on pancreatic *β*-cells from cytokine (IL-1)-induced damage has been shown through enhancing SIRT-1 expression and decreasing NF-ĸB activity [205]. 7-ketocholesterol (7-KC), a cholesterol oxidation product, can alter pancreatic *β*-cell activity in MIN6 cells. 7-KC reduced insulin production and accelerated the arrest of G0/G1, DNA damage, and interleukin-1 expression. Furthermore, it also caused *β*-cell senescence by blocking the SIRT-1/CDK4-Rb-E2F1 signalling pathway [206].

In a mouse model of T2DM, researchers discovered a novel chemical 5-(4-methylpiperazin-1-yl)-2-nitroaniline (6d) that inhibits SIRT-6, increases the expression of GLUT-1, lowers the blood glucose levels and has the potential to treat diabetes [207]. DM causes hepatic lipotoxicity, characterized by lipid buildup, excessive lipid peroxidation, pro-inflammation, low glutathione content, and elevated HDAC activity. Treatment with sodium acetate mitigated these changes by reducing HDAC activity and improving insulin sensitivity, indicating it may have therapeutic potential in treating diabetes-related liver problems [208].

2,4-dioxo-N-(4-(pyridin-3-yloxy)phenyl)-1,2,3,4-tetrahydroquinazoline-6-sulfonamide (SIRT6-IN-1), a SIRT-6 inhibitor, improved oral glucose tolerance in HFD fed mice. It also boosted the expression of the glucose transporters GLUT-1 and GLUT-4 in muscle, improved glycolytic pathway activity, and decreased plasma insulin, triglyceride, and cholesterol levels [209].

TSA inhibits class I, II HDACs, and SIRT-6 [210]. TSA and NaB increased the number of *β*-cells by increasing the formation of NGN3 + endocrine progenitors and altering endocrine subtype lineage choices on embryonic day 13.5 (E13.5) rat pancreas, and their use has been proposed to develop a new cell replacement therapy for T2DM [211]. The preculture of the *β*-cell line, INS-1 with TSA in the presence or absence of Interferon-α (IFN-α) and interleukin-1 (IL-1), had been shown to down-regulate iNOS expression with NO generation and death [212]. A recent study has shown that TSA inhibited the harmful effect of cytokines on *β*-cells but did not provide protection against cytotoxic activity. The effect of interleukin-1 (IL-1) on insulin-secreting INS 832/13 (INS) cells ability to secrete NO was examined, and it found that iNOS production and NO release were decreased in a concentration-dependent manner by the TSA. When TSA blocked IL-1*β*-mediated effects, histone H4 acetylation-mediated gene activation also increased. Rottlerin, a protein kinase C inhibitor, also increased histone H4 acetylation and reduced IL-1-induced NO release and iNOS production in INS cells, suggesting that rottlerin increases HATs activity and stimulates gene activation. Overall, the data support that PKCδ mediated signalling favours hypoacetylation of histones and results in IL-1*β*-induced metabolic dysfunction in isolated *β* cells [213].

The transcription factors SNAI1/SNAI2 work with the HDAC-i TSA to suppress the expression of the SLC2A5 gene, which codes for the high-affinity fructose transporter, GLUT5, linking fructose intake and diabetes [214].

**Fig. 7:**
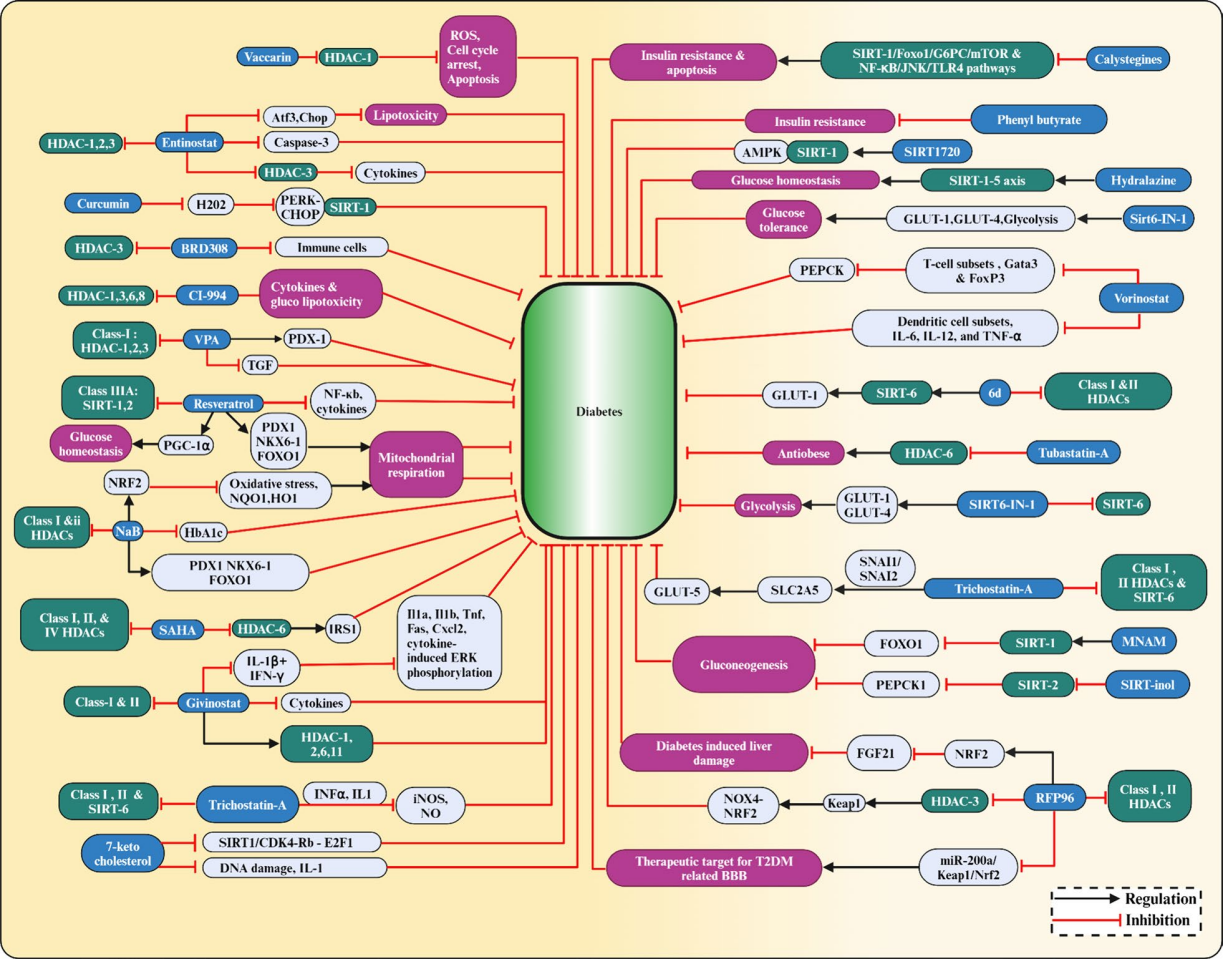
The regulatory roles of HDAC modulators in different cellular pathways, cell cycle, ROS, apoptosis, lipotoxicity, glucolipotoxicity, glucose homeostasis, mitochondrial respiration, gluconeogenesis, glycolysis, glucose tolerance, insulin resistance, *β*-cell development, and their contribution to diabetes. Black arrows represent regulation, while negative regulation is shown by red arrows with blunt ends

It has also been suggested that the SIRT-1/SIRT-5 axis is activated by hydralazine, improving metabolic homeostasis and mitochondrial function, which underlies its benefits in prolonging life and stress tolerance. Under high glucose and other stress situations, hydralazine also safeguards mitochondrial metabolism and function, restoring health and lifespan in C. elegans, suggesting a role in glucose homeostasis [215]. It was reported that maternal diabetes in vivo or high glucose in vitro increased histone 3 acetylation at lysine residues, H3K56, H3K14, H3K9, and H3K27, suspected substrates of SIRT-2 and SIRT-6 and these elevations were inhibited by SOD1 over-expression. Overexpression of SIRT-2 or SIRT-6 abolished high glucose-suppressed SIRT-2 or SIRT-6 expression and prevented acetylation of their histone substrates. The potent SIRT-uin activator called SRT1720 inhibited high glucose-induced histone acetylation and the formation of diabetes-induced neural tube defects (NTD). On the contrary, combining a pharmacological SIRT-2 inhibitor and a pan SIRT inhibitor mimicked the effect of high glucose-added histone acetylation and NTD induction. Thus, oxidative stress-induced SIRT-uin down-regulation that induces histone acetylation may be involved in diabetes-induced NTDs [216]. SRT1720 affects glucose homeostasis and the progression of metabolic diseases by activating AMPK and SIRT-1, suggesting another prospective therapeutic target for diabetes treatment [217]. Analysis of the effects of N1-methylnicotinamide (MNAM) on insulin resistance and glucose metabolism in obese mice with T2DM, as well as the regulatory mechanisms of the NAD-dependent deacetylase SIRT-1 (SIRT-1)/FOXO1) pathway, suggested that MNAM inhibits the gluconeogenesis pathway in the liver, increases SIRT-1 expression, and decreases acetylation of the FOXO1 protein, resulting in lower hepatic glucose production, improved insulin resistance and mitigating T2DM [218]. SIRT-inol, a SIRT-2 inhibitor, lowered the protein level of PEPCK1 and increased its acetylation, implying that SIRT-inol promotes the breakdown of PEPCK, which inhibits gluconeogenesis and diabetes [219]. Calystegines from Hyoscyamus albus give cytoprotection of HepG2 cells against insulin/glucose-induced insulin resistance and apoptosis by modulating the SIRT-1/Foxo1/G6PC/mTOR and NF-κB/JNK/TLR4 signalling pathways [220]. The role of HDAC modulators in various cellular pathways is shown in Fig. 7.

## Beyond the lab: insights from ongoing clinical trials on HDAC inhibitors in treating diabetes

These studies highlight the importance of characterizing epigenetic pathways involving HDAC regulation in DM in preclinical disease models and related clinical trials. So far, the FDA has approved four HDAC inhibitors, vorinostat, romidepsin, panobinostat, and belinostat, for cancer and cardiovascular disease treatments [221]. Although HDAC-i is useful in reducing macrovascular problems such as coronary heart disease, diabetic nephropathy, and diabetic retinopathy, none have reached clinical practice yet [222]. However, few clinical trials have been conducted using HDAC-i to treat diabetes. The University Health Network in Toronto evaluated the impact of buphenyl (phenylbutyrate) on fatty acid-induced impairment of glucose-stimulated insulin secretion in healthy males (phase IV single-blind clinical trial identifier-NCT00533559). The results of this tiny trial, which involved eight patients, were published in March 2010 and showed that phenylbutyrate, when taken orally for two weeks at a dose of 7.5 g per day, improves insulin resistance and blood sugar levels in humans [223]. Lysine deacetylase inhibitors protect *β*-cells from inflammatory destruction in vitro and are promising immunomodulators in insulin-producing *β*-cells. The NF-κB subunit p65's hyperacetylation was linked to these effects. Vorinostat administration raised the regulatory T-cell subsets and their transcription factors Gata3 and FoxP3 while decreasing the frequency of inflammatory dendritic cell subsets and associated cytokines IL-6, IL-12, and TNF-α. Givinostat reduced IL-1*β* + IFN-*γ*–induced proinflammatory Il1a, Il1b, Tnf, Fas, and Cxcl2, as well as cytokine-induced ERK phosphorylation [224]. Recently, a randomized phase II clinical trial with 282 patients suffering from diabetic peripheral neuropathy was administered with HDAC-6 inhibitor, Ricolinostat, over 12 weeks, and results are still awaited [225]. Although the above clinical trials demonstrated the effectiveness of HDAC-i in mitigating DM, it is crucial to note that the use of HDAC-i in diabetic complications requires further studies to understand their side effects. Therefore, most HDAC-i have enormous potential to fight diabetes with further drug research and human clinical trial advancement.

## Conclusion

Clinical epigenetics is still a developing field, although some biomarkers for different diseases are being identified. Most of this progress is mainly in cancer, and metabolic diseases are still in their infancy. HDACs are critical components of cellular and molecular pathways needed for glucose homeostasis, pancreatic *β*-cell development, *β*-cell maintenance, *β*-cell protection, and insulin expression and secretion. The gene ontology and functional analysis revealed the specific HDACs, associated gene targets, and their link to T2DM, which shed light on changes in the regulatory interactions that contribute to the development of T2DM. The different types of HDAC-i and their implication in clinical studies for diabetes treatment are only beginning to be understood. The fact that the HDAC-i inhibitory processes are poorly understood and are frequently linked to adverse side effects implies further research is needed on their application in managing T2DM. Since HDACs have therapeutic potential for treating diabetes, finding effective HDAC-i against diabetes requires extensive research in drug development and clinical research.

### Prospects on the horizon: envisioning future developments

Diabetes has become a global health concern, and a comprehensive understanding of it in connection to HDACs and HDACi will enable better treatment. The emerging evidence from animal models suggests that HDAC-i can protect and help regenerate pancreatic *β*-cells by restoring and maintaining *β*-cell function. These molecules have proven to contribute to glucose homeostasis by promoting the survival and replication of insulin-producing *β*-cells. During preclinical trials, HDAC-i emerged as a promising drug candidate that improves insulin sensitivity, glucose uptake by cells and overall glycemic management in people with diabetes through its anti-inflammatory and antioxidant activities, altered histone acetylation state, and changes in gene expression profiles. However, there were also adverse effects and challenges. One main difficulty is the specificity and the level of change we need. This is crucial as the changes in multifactorial diseases such as T2DM are specific for each patient. So, there is a need for strategies that could be targeted based on epigenomic profiles. This also should be highly tissue and cell-specific. Further HDAC-specific HDACi are needed rather than general HDACi, which target more than one. Another treatment strategy called combination therapy can be considered, where HDAC-i with other antidiabetic drugs can work in synergy for better glycaemic control. More extensive computational research in drug discovery is also needed to understand HDAC-i interactions with HDACs, which would aid in designing novel HDAC-i with greater specificity and potency. It is vital to emphasize that HDAC-i development and translation for DM treatment are still in their early stages. Therefore, research efforts are essential to decipher their mechanisms, optimize the drug administration process, and assess long-term safety by conducting clinical trials to determine their efficacy in combating T2DM.

## Data Availability

Not applicable.

## Abbreviations

6d: 5-(4-Methylpiperazin-1-yl)-2-nitroaniline
7-KC: 7-Ketocholesterol
ALDOA: Aldolase
AMPK: AMP-activated protein kinase
ART: Artesunate
ATF: Activating transcription factor
ATX: Autotaxin
CHOP: CCAAT/enhancer-binding protein homologous protein
CI-994: N-acetyldinaline
FOX P3: Forkhead box P3
FOXO: Forkhead box gene
GABA: Gamma-aminobutyric acid
GCR: Glucocorticoid receptor
GKRP: Glucokinase regulatory protein
GLP-1: Glucagon-like peptide-1
GLUT4: Glucose transporter 4
HAT: Histone acetyltransferase
HDAC: Histone deacetylase
HDACi: HDAC inhibitors
HFD: High-fat diet
HO1: Heme oxygenase 1
IFN-α: Interferon-α (IFN-α)
IL-1: Interleukin-1
IRS: Insulin receptor substrate 1
JNK: Jun N-terminal kinase
KDACi: Histone lysine deacetylase inhibitors
LDH: Lactate dehydrogenase
MafA: V-maf musculo aponeurotic fibrosarcoma oncogene A
MGCD0103: Mocetinostat
NaB: Sodium butyrate
NeuroD1: Neurogenic differentiation 1
NFAT: Nuclear factor of activated T cells
NF-κB: Nuclear factor kappa B
NQO1: NAD(P)H dehydrogenase quinone 1
NRF2: Nuclear factor erythroid 2-related factor 2
NTD: Neural tube defect
Pax4: Activated paired box-4
PDX1: Pancreatic duodenal homeobox 1
PFK-1: Phosphofructose kinase 1
PGK-1: Glucose-6-phosphate isomerase
p-PERK: ER-resident protein kinase
PRMT6: Protein arginine methyltransferase
PTP1B: Protein tyrosine phosphatase 1b
QTL: Quantitative trait locus
SAHA: Suberanilohydroxamic acid
SFG: Sulfated fucogalactan
SIRT6-IN-1: 2,4-Dioxo-N-(4-(pyridin-3-yloxy)phenyl)-1,2,3,4-tetrahydroquinazoline- 6-sulfonamide
SIRTs: Sirtuins
STZ: Streptozotocin
T2DM: Type2 diabetes mellitus
Tph1: Tryptophan hydroxylase 1
TSA: Trichostatin A
Txnip: Thioredoxin-interacting protein
VAC: Vaccarin
VPA: Valproic Acid
